# Microbiota alter metabolism and mediate neurodevelopmental toxicity of 17β-estradiol

**DOI:** 10.1038/s41598-019-43346-9

**Published:** 2019-05-08

**Authors:** Tara R. Catron, Adam Swank, Leah C. Wehmas, Drake Phelps, Scott P. Keely, Nichole E. Brinkman, James McCord, Randolph Singh, Jon Sobus, Charles E. Wood, Mark Strynar, Emily Wheaton, Tamara Tal

**Affiliations:** 1 Oak Ridge Institute for Science and Education, Oak Ridge, TN USA; 2 U.S. EPA/ORD/NHEERL/RCU, RTP, NC USA; 3 U.S. EPA/ORD/NHEERL/ISTD, RTP, NC USA; 4 U.S. EPA/ORD/NERL/SED, Cincinnati, OH USA; 5 U.S. EPA/ORD/NERL/EMMD, RTP, NC USA; 6 Present Address: Boehringer Ingelheim Pharmaceuticals Inc., Ridgefield, CT USA

**Keywords:** Toxicology, Applied microbiology

## Abstract

Estrogenic chemicals are widespread environmental contaminants associated with diverse health and ecological effects. During early vertebrate development, estrogen receptor signaling is critical for many different physiologic responses, including nervous system function. Recently, host-associated microbiota have been shown to influence neurodevelopment. Here, we hypothesized that microbiota may biotransform exogenous 17-βestradiol (E2) and modify E2 effects on swimming behavior. Colonized zebrafish were continuously exposed to non-teratogenic E2 concentrations from 1 to 10 days post-fertilization (dpf). Changes in microbial composition and predicted metagenomic function were evaluated. Locomotor activity was assessed in colonized and axenic (microbe-free) zebrafish exposed to E2 using a standard light/dark behavioral assay. Zebrafish tissue was collected for chemistry analyses. While E2 exposure did not alter microbial composition or putative function, colonized E2-exposed larvae showed reduced locomotor activity in the light, in contrast to axenic E2-exposed larvae, which exhibited normal behavior. Measured E2 concentrations were significantly higher in axenic relative to colonized zebrafish. Integrated peak area for putative sulfonated and glucuronidated E2 metabolites showed a similar trend. These data demonstrate that E2 locomotor effects in the light phase are dependent on the presence of microbiota and suggest that microbiota influence chemical E2 toxicokinetics. More broadly, this work supports the concept that microbial colonization status may influence chemical toxicity.

## Introduction

Essentially all known multicellular organisms are naturally colonized by a diverse array of microbial communities comprised of bacteria, viruses, archaea, fungi and protozoa. Current estimates suggest that the adult human gastrointestinal tract alone harbors more than 100 trillion bacteria from over 1,000 different species^1^. The metagenome of these gut microbiota encodes 100–150 times more genes than the human genome, with rapid plasticity and unique biological functions^2^. Microbiota can interact with their hosts directly or through the production of various microbial products, such as extracellular enzymes and cell wall components^3^ to influence immune and nervous systems, metabolism, and behavior^4–6^. Microbiota can also interact with drugs and environmental chemicals in ways that can shift their health effects or toxicity profiles^7–9^. Such findings indicate that host microbiota can be an important determinant of both homeostasis and disease risk.

Neurodevelopmental disorders like autism-spectrum disorder and attention-deficit/hyperactivity disorder have increased in prevalence over the last decade^10^. Emerging evidence indicates that microbiota may mediate or influence neurologic development. During early life, different stages of brain development coincide with the establishment and development of the gut microbiome^11^. Recent studies have associated some neurodevelopmental disorders with altered microbiota profiles^12^, and experiments that use axenic (microbe-free) animals or antibiotics to deplete host-associated microbiota indicate that microbiota influence key aspects of early neurodevelopment. Affected processes include formation of the blood-brain barrier^13^, myelination^14^, neurogenesis^15^, neurotransmitter levels^16^, and behavior^17–19^. Collectively, these interactions are driven by the microbiota-gut-brain axis, which involves bidirectional communication between intestinal microbiota and the central nervous system.

The increased prevalence of neurodevelopmental disorders also corresponds with increased exposure to endocrine disrupting chemicals (EDCs) worldwide^20^. Endogenous steroid hormones such as 17β-estradiol (E2) are critical for nervous system development and mediate endpoints such as neuronal survival, dendritic branching, synaptic patterning, and axonal projections^21^. Increasing concentrations of estrogens in the environment have led to widespread public health concerns, especially given the sensitivity of the developing organism to even low micromolar or nanomolar concentrations of estrogens^22^. Recent studies suggest that exposure to estrogenic chemicals during sensitive windows of early brain development can disrupt key signaling pathways and result in adverse neurological effects^23–25^. Intestinal microbiota play a significant role in the uptake and metabolism of estrogens^26,27^. Through processes such beta-glucuronidation, gut microbiota can affect chemical enterohepatic circulation and excretion and potentially alter endogenous serum estrogen concentrations^28–30^. However, it is currently unknown whether microbiota can interact with exogenous estrogens to mediate neurodevelopmental effects.

Zebrafish are an alternative animal model commonly used in toxicology to examine effects of contaminant exposure. More recently, zebrafish have also become an established model for studying host-microbiota interactions^19,31,32^. Approximately 70% of human protein-coding genes are similar to those in zebrafish and zebrafish contain counterparts for 84% of human disease-associated genes^33^. Advantages of the model include their rapid development, small size, transparency, and capability to perform complex motor behaviors. Development of distinct neural circuits generate the earliest behaviors in zebrafish, which serve as functional readouts of nervous system development^34^. Similar to other vertebrates, zebrafish have complex resident microbial communities with a large majority of microbiota concentrated in the intestinal tract^35,36^. However, zebrafish microbiota are typically dominated by Proteobacteria^37^, while mice and humans contain more Bacteroidetes and Firmicutes^35^, differences that are likely driven by diet, the surrounding environment, and host factors^35,38^. While microbial composition varies across species, more work is needed to understand whether metagenomic function is conserved between zebrafish and mammals. Our laboratory and others have recently established methods to generate axenic or colonized zebrafish^19,39,40^, which can be combined with various toxicity assays to evaluate microbiota interactions with environmental chemicals.

In this study, we investigated whether microbial colonization status interacts with E2 to influence locomotor activity and chemical metabolism in zebrafish. We first characterized the effects of exogenous E2 exposure on locomotor activity in colonized zebrafish as a functional measure of nervous system development and investigated changes in host-associated microbial community structure and predicted metagenomic function. We then assessed colonization status-dependent changes in locomotor activity using both colonized and axenic zebrafish. Finally, we conducted targeted and non-targeted mass spectrometry to measure internal E2 dose and identify colonization status-specific metabolite profiles. To our knowledge, this is the first study to demonstrate light phase specific locomotor effects that require microbial colonization following exposure to an exogenous chemical. These findings also show, using a whole organism model, that colonization status influences metabolism of a key sex steroid involved in endocrine signaling.

## Results

### Observed overt toxicity and locomotor effects following developmental exposure to E2

**Figure 1:**
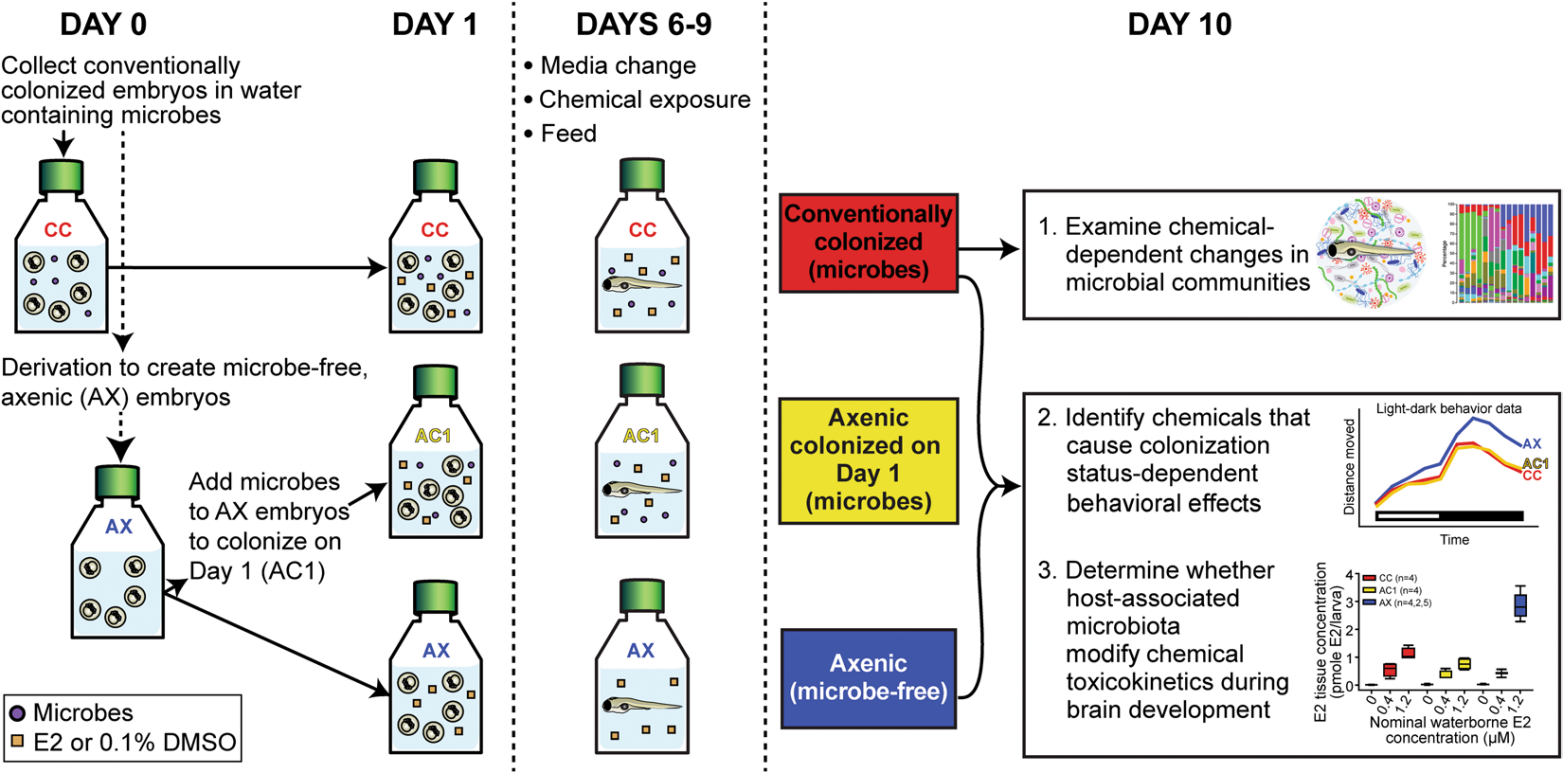
Experimental design. Endpoints assessed on day 10 in larval zebrafish following exposure to exogenous E2 on days 1, 6, 7, 8, and 9 are shown. Experiments were conducted in conventionally colonized (CC), axenic colonized on day 1 (AC1) and/or axenic (AX; microbe-free) zebrafish cohorts.

**Figure 2:**
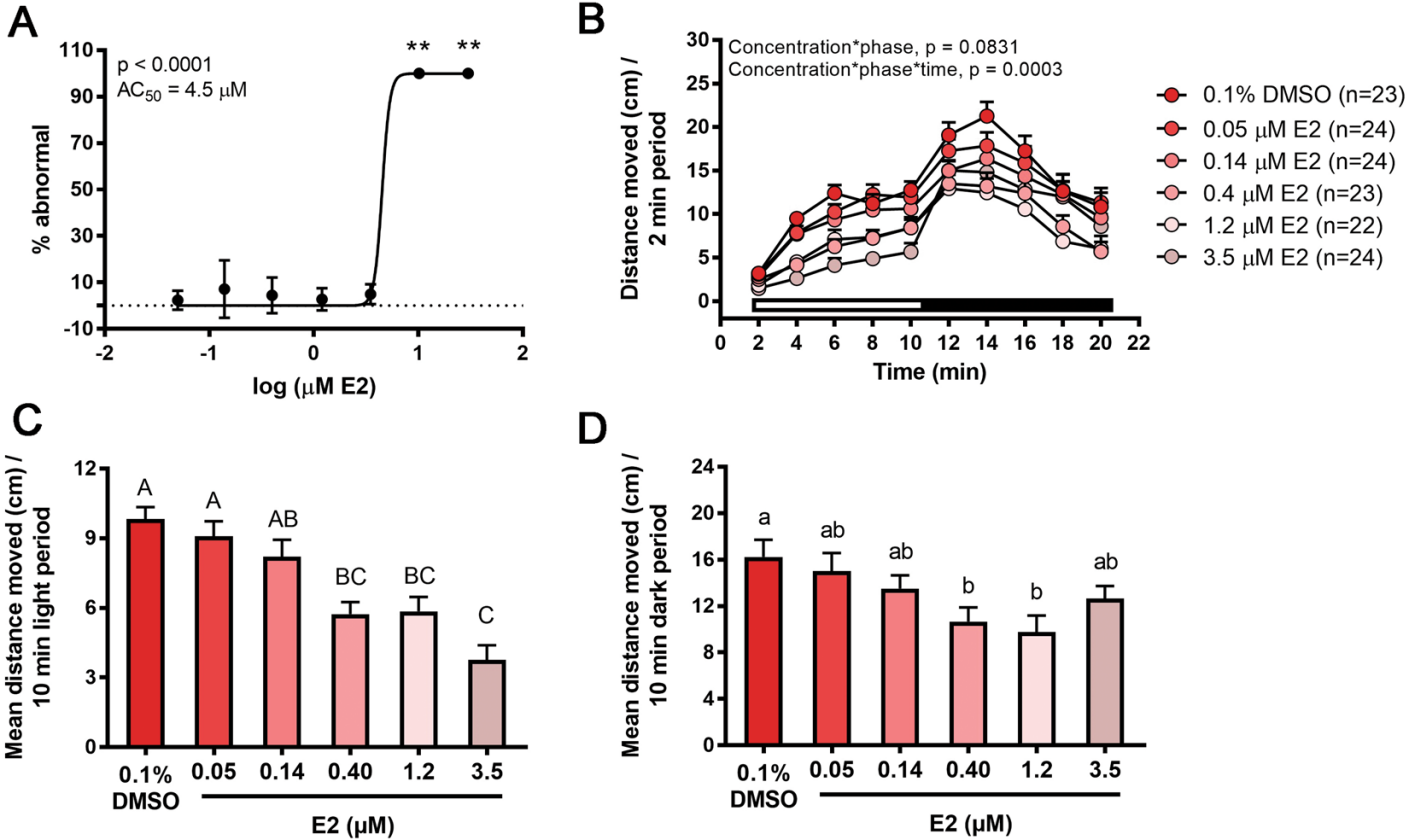
Conventionally colonized zebrafish developmentally exposed to non-teratogenic E2 concentrations are hypoactive. (**A**) Percent abnormal zebrafish larvae are shown. Non-linear regression was performed using the log(agonist) *vs*. normalized response–Variable slope equation in GraphPad Prism. Asterisk indicates significant difference from respective DMSO vehicle control group (**p < 0.0001). n = 3 replicate flasks with 15 larvae per flask. Standard error bars are shown. (**B**) Movement over 20-minute testing phase and mean distance moved in the (**C**) light or (**D**) dark period following developmental E2 exposure are shown. White and black bars indicate light and dark phases, respectively. n = 23–24 larvae per treatment.

To examine whether microbiota can influence behavior or metabolism of E2 during development, we generated conventionally colonized, axenic, or axenic colonized on day 1 larvae^19^ (Fig. 1). Conventionally colonized zebrafish were first exposed continuously in a semi-static system to E2, and survival and morphology were assessed on day 10 to identify the no observed effect concentration (NOEC) for follow-up studies. By 10 days post-fertilization (dpf), all larvae in the 10.2 µM exposure group were abnormal (malformed or severely moribund) or dead, and 100% mortality was observed in the 30.0 µM exposure group (Fig. 2A). All other treatment groups up to 3.5 µM contained ≥92% morphologically normal larvae resulting in a calculated AC_50_ value (concentration at 50% of the maximal effect) of 4.5 µM (Fig. 2A). Locomotor activity was subsequently assessed as a functional measure of nervous system development in conventionally colonized larvae exposed to non-teratogenic concentrations of E2. Concentration-dependent locomotor hypoactivity was observed in both the light and dark phases (Fig. 2B–D). Significant decreases in activity were observed at 0.4, 1.2, and 3.5 µM E2 in the light phase, and at 0.4 and 1.2 µM in the dark phase (Fig. 2C,D). At 3.5 µM in the dark, activity was similar to DMSO vehicle controls (Fig. 2D).

### E2 exposure does not impact zebrafish-associated microbial communities

**Figure 3:**
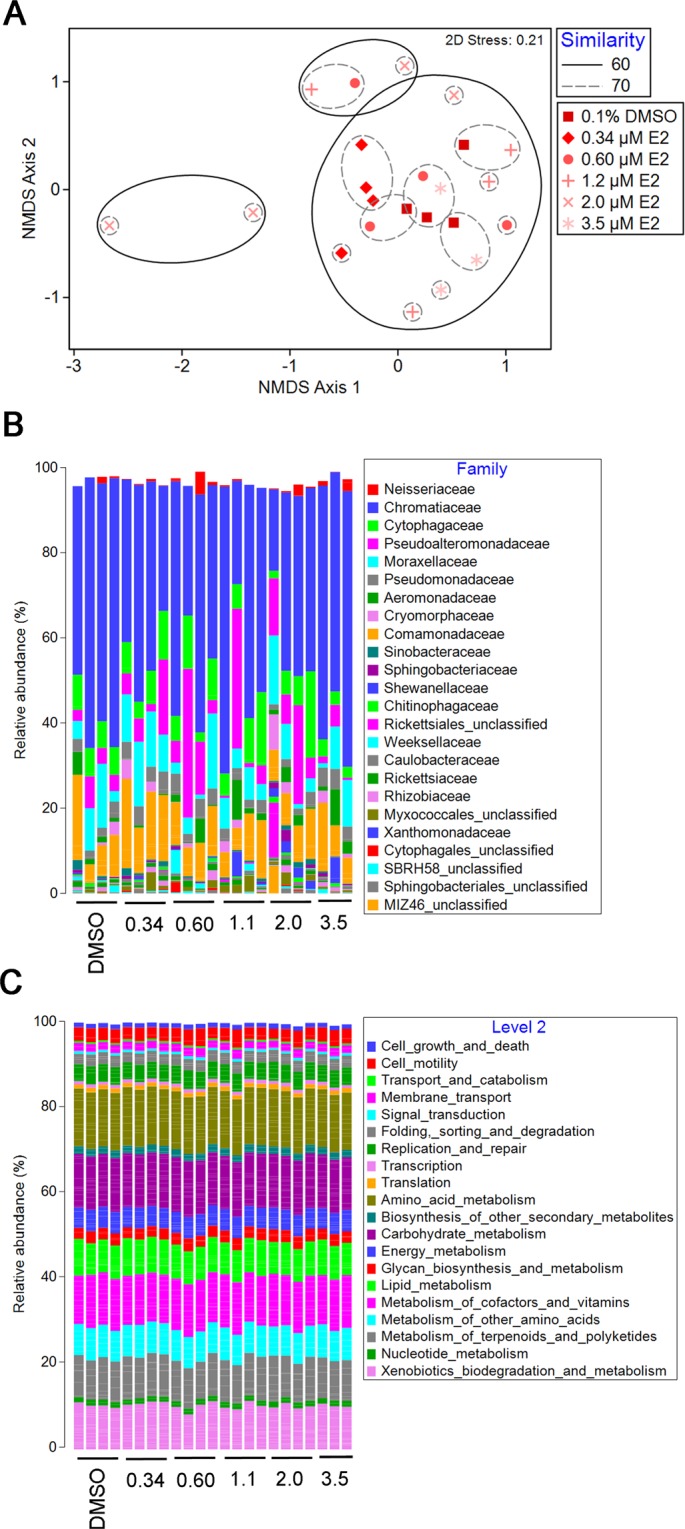
E2 exposure does not impact community structure or predicted function of zebrafish-associated microbiota. 16S rRNA gene sequencing was performed on 10 dpf larvae developmentally exposed to E2, and PICRUSt was used to predict metagenomic function. Non-metric dimensional scaling (NMDS) ordination plots are shown for microbial community structure. The Bray-Curtis similarity index was used to assign percent similarity values. Analysis of similarities (ANOSIM) ordered R (OR) statistic is shown. Percent relative abundances of (**B**) family-level taxa or (**C**) Level 2 KEGG predicted functions in 10 dpf larvae are shown. n = 4 biological replicates with 10 larvae per replicate.

To identify whether altered behavior following E2 exposure co-occurred with changes in microbial community structure or predicted microbial community function, conventionally colonized zebrafish were exposed to five non-teratogenic E2 concentrations, and 16S rRNA gene sequencing was used to identify operational taxonomic units (OTUs) present in each sample. Non-metric multidimensional scaling (NMDS) analysis revealed that global compositional profiles of microbiota did not cluster by E2 treatment (ANOSIM OR = 0.064) (Fig. 3A). Bray-Curtis similarity indices for all between-treatment group comparisons (e.g. DMSO vehicle control *vs*. 0.34 µM E2) were similar and ranged on average from 55.8–68.1% (Fig. S1). Taxonomic analysis revealed that Proteobacteria was the most abundant phylum at all concentrations tested (data not shown). In addition, 24 bacterial families captured >95.6% of all OTUs identified represented, and the relative abundances of family-level taxa were similar across all DMSO control and E2-exposed larvae (Fig. 3B). E2 exposure also did not impact any alpha diversity metrics including total number of species, Margalef’s species richness index, species evenness, Shannon’s diversity index, or Simpson’s diversity (Fig. S2). Relative abundances of PICRUSt-generated Level 2 KEGG predicted functions were similar across all treatment groups (Fig. 3C). To assess whether E2-exposure impacted function at a more granular level, linear discriminant effect size (LEfSe) analysis was subsequently used to identify differentially abundant Level 3 KEGG functions (Table S1). All 11 identified functions were not significantly altered in E2-exposed larvae relative to DMSO vehicle controls (Fig. S3).

### E2-induced locomotor effects in the light phase are dependent on microbiota

**Figure 4:**
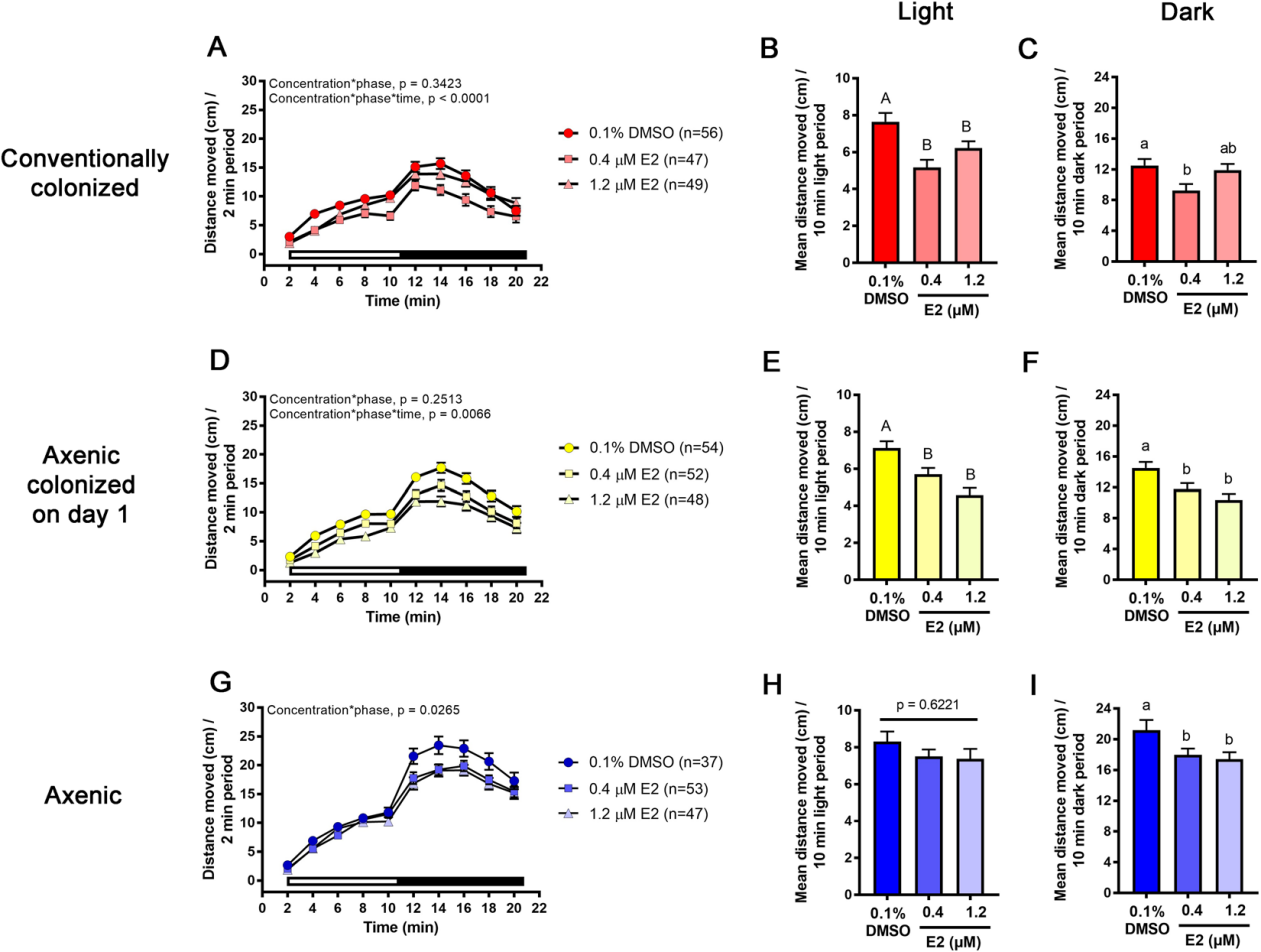
Locomotor hypoactivity is observed in the light phase in E2-exposed colonized zebrafish only. (**A,D,G**) Movement over 20-minute testing period, (**B,E,H**) movement over 10 min light phase, or (**C,F,I**) movement over 10 min dark phase are shown. White and black bars indicate light and dark phases, respectively. Data for conventionally colonized (**A**–**C**), axenic colonized on day 1 (**D**–**F**), and axenic (**G**–**I**) larvae are shown. If a significant 3- or 2-way interaction was observed using a linear mixed effect repeated measures model, subsequent Tukey pairwise comparisons were made. Different letters indicate significant differences in the light (uppercase) or dark (lowercase) phases (p < 0.05). n = 23–24 larvae per treatment.

To examine whether hypoactivity observed in conventionally colonized larvae following E2 exposure (Fig. 2B,C) was related to microbial colonization of the host, three cohorts of zebrafish (conventionally colonized, axenic colonized on day 1, and axenic)^19^ (Fig. 1) were continuously exposed to 0.4 or 1.2 µM E2. As expected, DMSO control axenic zebrafish were hyperactive in the dark phase relative to conventionally colonized or axenic colonized on day 1 zebrafish (p < 0.0001, Figs 4A–I and Fig. S4). At 10 dpf, conventionally colonized larvae exhibited significant hypoactivity in the light phase at 0.4 and 1.2 µM E2 (p < 0.00001 and p = 0.0407, respectively, Fig. 4B), similar to the initial behavior experiment in conventionally colonized larvae (Fig. 2BC). In the dark phase, significant hypoactivity was observed at 0.4 µM E2, but not 1.2 µM E2 (p = 0.0043 and p = 0.5862, Fig. 4C). In axenic colonized on day 1 larvae, significant hypoactivity was observed in both the light and dark phases at 0.4 and 1.2 µM E2 (light: p = 0.0033 and p < 0.0001, respectively, dark: p = 0.0259 and p = 0.0004, respectively) (Fig. 4E,F). In contrast to colonized larvae, axenic larvae exhibited no significant change in locomotor activity during the light phase compared to DMSO vehicle controls (Fig. 4H). In the dark, axenic larvae behaved like colonized larvae exhibiting locomotor hypoactivity at 0.4 and 1.2 µM E2 (p = 0.0041 and p = 0.0026, respectively) (Fig. 4I).

### Axenic zebrafish contain more parent E2 than colonized zebrafish

**Figure 5:**
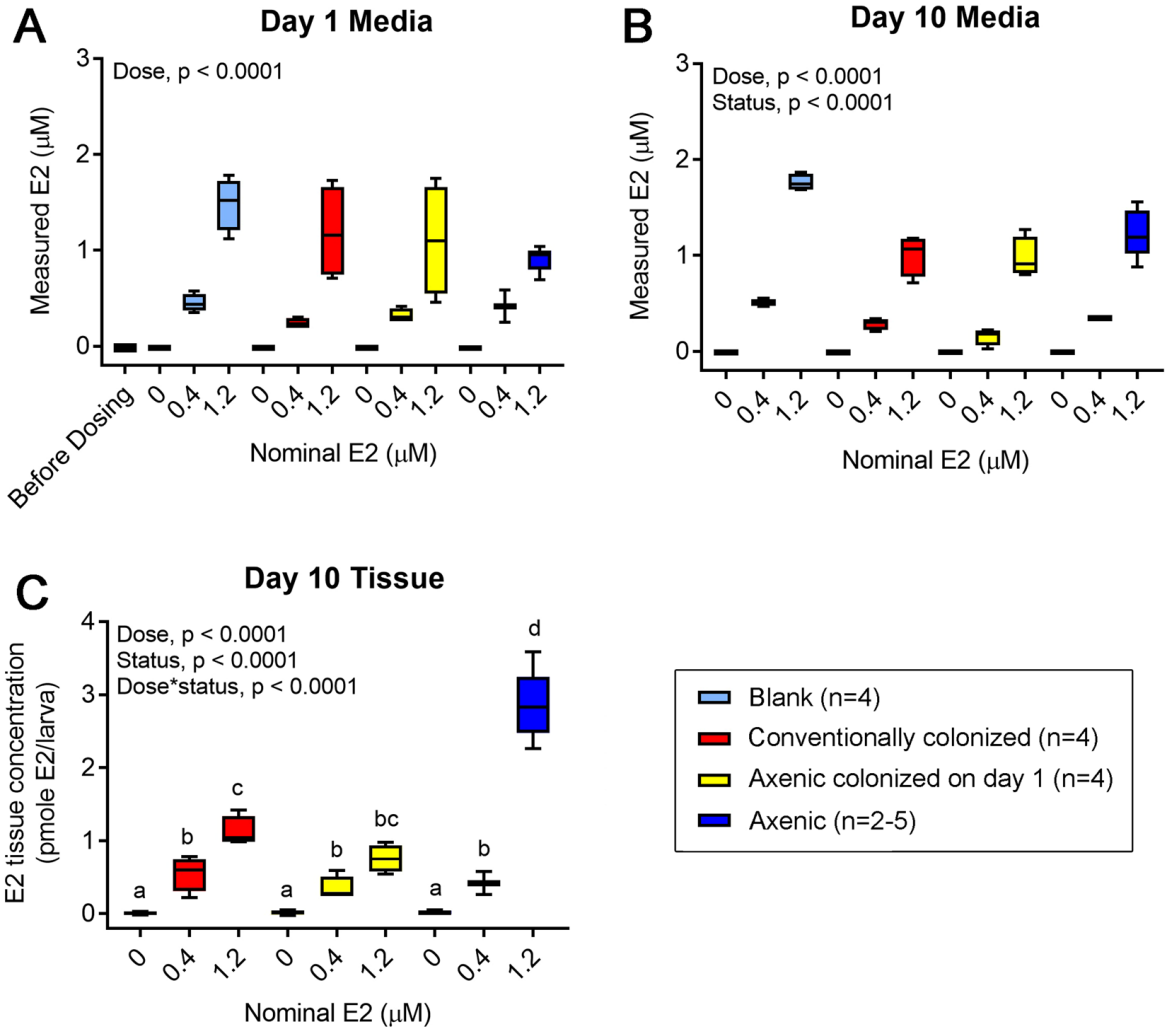
Axenic zebrafish contain higher concentrations of parent E2. Exposure media and whole zebrafish tissue were analyzed by LC-MS/MS. Concentrations of parent E2 in (**A**) exposure media on day 1, (**B**) exposure media on day 10 or (**C**) zebrafish tissue on day 10. A concentration of 0 µM refers to 0.1% DMSO vehicle controls. Blank (fish-free) flasks contained no zebrafish and were treated like axenic flasks throughout the duration of the experiment. If a significant 2-way interaction was observed using multiple linear regression models, subsequent pairwise comparisons (Bonferroni correction, p < 0.05) were made. n = 2–5 (10 larvae per biological replicate).

To compare measured E2 concentrations in exposure media and whole-body tissue homogenates (that included intestinal tracts) obtained from conventionally colonized, axenic colonized on day 1, and axenic larvae continuously exposed to 0.4 or 1.2 µM E2, samples were collected and analyzed using targeted chemistry techniques. Immediately following dosing on day 1, measured E2 media concentrations were similar across colonization statuses and ranged from 0.242–0.453 µM and 0.910–1.485 µM for the 0.4 and 1.2 µM E2 exposure groups, respectively (Dose, p < 0.0001, Fig. 5A and Table S2). In day 10 media samples, significant main effects of dose (p < 0.0001) and colonization status (p < 0.0001) were observed (Fig. 5B). Average measured media concentrations ranged from 0.155–0.509 µM for the 0.4 µM E2 group, and 0.975–1.76 µM for the 1.2 µM E2 group (Fig. 5B and Table S2). Individual doses were not compared across colonization status because no significant interaction between dose and colonization status was observed. Analysis of day 10 zebrafish tissue revealed distinct colonization-dependent differences in measured E2 concentration in zebrafish exposed to 1.2 µM E2. In addition to significant main effects of dose (p < 0.0001) and colonization status (p < 0.0001), a significant interaction between these two terms was observed (p < 0.0001), indicating that the pattern of measured internal E2 doses across all concentrations tested differed by colonization status (Fig. 5C). Subsequent pairwise comparisons revealed that following exposure to a nominal concentration of 1.2 µM E2, measured E2 tissue doses in axenic larvae were 2.5–3.7 times higher than in conventionally colonized or axenic colonized on day 1 larvae (Fig. 5C and Table S2). At a nominal concentration of 0.4 µM E2, tissue doses were comparable across colonization status (Fig. 5C and Table S2).

### Axenic zebrafish contain higher abundances of some E2 metabolites

**Figure 6:**
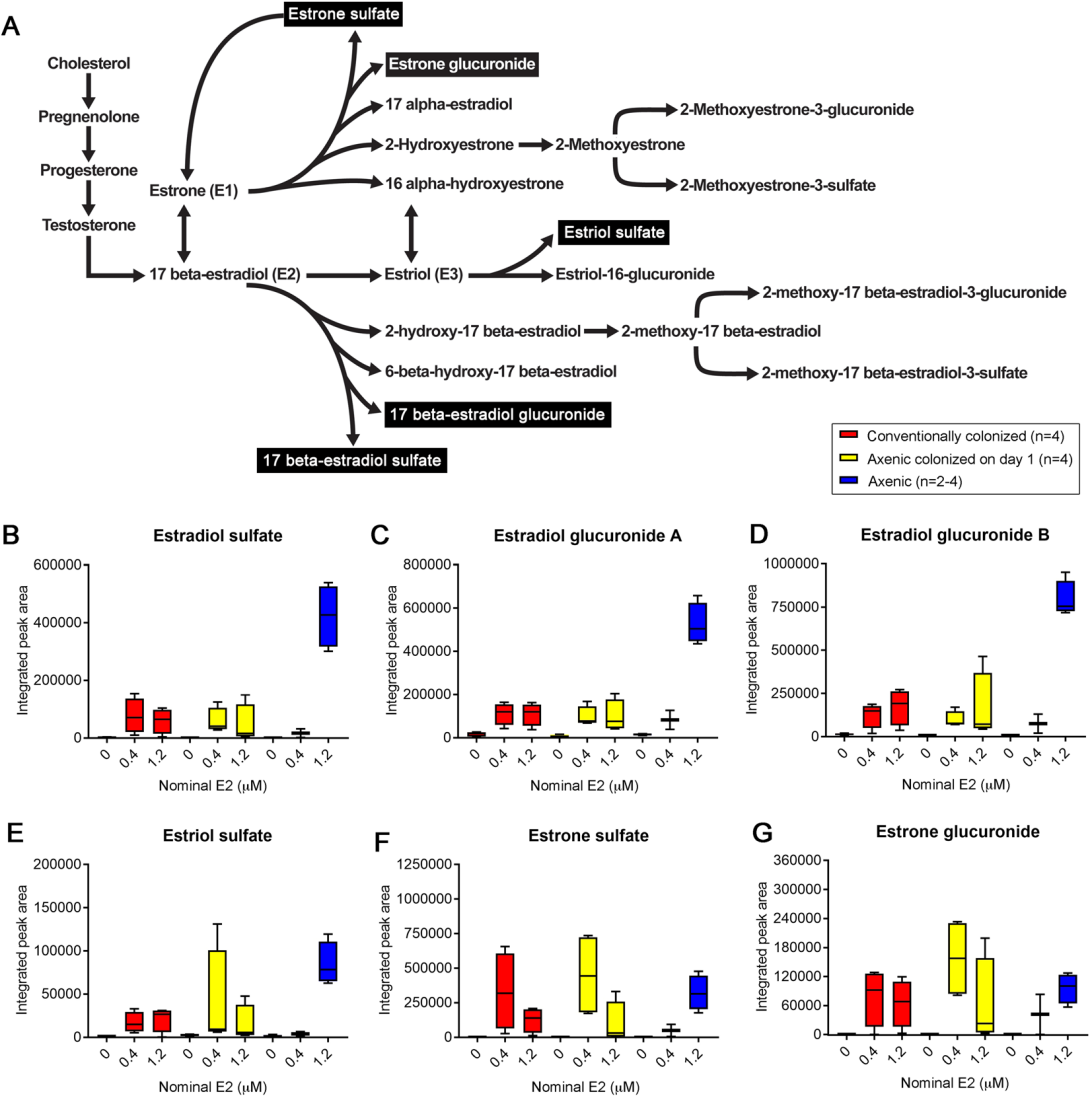
Axenic zebrafish contain higher concentrations of some putative E2 metabolites. LC*-*MS was performed on whole zebrafish tissue. (**A**) E2 metabolism reference schematic is shown. Black boxes indicate putative E2 metabolites identified in this study. Boxplots with integrated peak area for (**B**) estradiol sulfate, (**C**) estradiol glucuronide A, (**D**) estradiol glucuronide B, (**E**) estriol sulfate, (**F**) estrone sulfate and (**G**) estrone glucuronide in conventionally colonized, axenic colonized on day 1, and axenic larvae are shown. A concentration of 0 µM refers to 0.1% DMSO vehicle controls. Spearman rank correlation coefficients were calculated to assess the relationships between integrated peak areas for each detected E2 metabolite and measured E2 tissue concentrations from targeted analysis (Table 1). n = 2–4 (10 larvae per biological replicate).

As a hypothesis-generation strategy, a non-targeted approach was initially selected to identify any chemical features (e.g. E2 metabolites or microbial metabolites) that may be altered in conventionally colonized, axenic colonized on day 1, or axenic zebrafish following exposure to 0.4 or 1.2 µM E2. Following initial feature filtering, 94 features were retained in the dataset (Fig. S5). Comparison of features that met fold-change threshold criteria (≥2-fold) *within* colonization status (e.g. axenic 0.1% DMSO *vs*. axenic 1.2 µM E2) yielded numerous significant features in all three cohorts (Fig. S6A–C). *Between*-status comparisons at 1.2 µM E2 (e.g. conventionally colonized 1.2 µM to axenic 1.2 µM E2) revealed that 33 features were concordant (i.e. increased or decreased) in both conventionally colonized and axenic colonized on day 1 larvae compared to axenic larvae. Of these 33 features, 26 were significantly decreased and seven were significantly increased (Fig. S7A–C and Table S3). However, using this stringent filtering approach, only one feature was identified as a predicted E2 metabolite (E2 sulfate) (Fig. 6B), and other differentially represented unknown metabolites were not identified. Therefore, to specifically identify additional E2 metabolites that were differentially represented following exposure to the parent compound, the raw LC-MS data were screened for known metabolites in the E2 metabolism pathway (Fig. 6A). Five chemicals consistent with known metabolites were tentatively identified: E2 sulfate, E2 glucuronide (A and B, two isomers), estriol (E3) sulfate, estrone (E1) sulfate, and E1 glucuronide (Fig. 6A, Table 1). Integrated peak areas for all metabolites were significantly correlated (p < 0.0001) with measured E2 tissue concentrations. The strongest correlations were observed for E2 sulfate (r = 0.84) and the two isomers of E2 glucuronide (r = 0.87 and 0.90) (Table 1). Boxplots for these E2 metabolites (Fig. 6B–D) show very similar trends to that observed for parent E2 concentrations (Fig. 5C). Specifically, peak areas of these metabolites were noticeably elevated in the axenic 1.2 μM E2 group. Correlations were less pronounced for E3 sulfate (r = 0.73), E1 sulfate (r = 0.69), and E1 glucuronide (r = 0.69) (Table 1), with boxplots showing elevated peak areas across colonization status (Fig. 6E–G).

**Table 1:** Putative estradiol metabolites identified in 10 dpf zebrafish following non-targeted mass spectrometric analysis.

Putative Compound	Neutral Mass	Empirical mass-to-charge ratio (m/z)	Retention time (min)	Predicted Molecular Formula	Spearman correlation coefficient^a^ (p-value)
Estradiol sulfate	352.1344	351.1266	6.4	C_18_H_24_O_5_S	0.84 (<0.0001)
Estradiol glucuronide A	448.2097	447.2019	6.2	C_24_H_32_O_8_	0.87 (<0.0001)
Estradiol glucuronide B	448.2097	447.2019	6.7	C_24_H_32_O_8_	0.90 (<0.0001)
Estriol sulfate	368.1361	367.1283	6.8	C_18_H_24_O_6_S	0.73 (<0.0001)
Estrone sulfate	350.1190	349.1112	6.9	C_18_H_22_O_5_S	0.69 (<0.0001)
Estrone glucuronide	446.1940	445.1862	6.8	C_24_H_30_O_8_	0.69 (<0.0001)

^a^Correlations between peaks areas of putative E2 metabolites and measured E2 tissue concentrations.

## Discussion

Recent work suggests that estrogens and host-associated microbiota, particularly those within the intestinal tract, to influence a variety of health outcomes including brain function, behavior, obesity, and cancer^28^. In this study, we used colonized and axenic zebrafish as an experimental system to characterize the effects of E2, a potent estrogen receptor agonist, on microbiota composition and predicted metagenomic function. In addition, we asked whether E2 exposure impacted locomotor activity in a colonization status-dependent manner. We report that E2 did not impact microbial community structure or predicted function. However, E2 exposure did result in significant hypoactivity in the light phase in colonized larvae only. Measured parent E2 concentrations were ~3 times higher in axenic relative to colonized zebrafish. Predicted E2 metabolites E2 sulfate and E2 glucuronide were also higher in axenic relative to colonized zebrafish. In contrast, E3 sulfate, E1 sulfate and E1 glucuronide were similar in colonized and axenic larvae. To our knowledge, this is the first study to demonstrate that microbiota interact with an exogenous chemical to influence light phase locomotor effects. Taken together, these data support the concept that microbiota and environmental chemicals can interact to influence host physiology.

In mammals, the estrogen signaling pathway has been implicated as an important regulator of various cognitive and behavioral responses. For example, low estrogen levels have been associated with a decline in memory-related functions in humans and animal models^41,42^. Similar to the hypoactivity data presented here, exposure to E2 in ovariectomized mice resulted in locomotor hypoactivity in a light/dark transition test^43^. The same study also reported increased time spent in the outer zones in an open field test^43^. These two widely used mammalian tests measure anxiety-like behavior, suggesting that, in animals lacking endogenous estrogens, E2 exposure increased anxiety-like behavior and heightened fear response, both of which are acquired adaptive survival behaviors^44^. In a female rat study, E2 treatment during the neonatal period resulted in improved learning and enhanced memory performance in adulthood^45^. These effects were hypothesized to be related to E2 effects on γ-aminobutyric acid (GABA) receptor expression and function in the adult brain^45^. Together, these studies suggest that exogenous E2 exposure has widespread effects on nervous system function in mammalian models.

The circuits that underlie the patterned development of stereotypical locomotor behaviors are highly regulated, and it is now understood that microbiota play a critical role in the development of many normal locomotor phenotypes and regulate neurophysiological behaviors through alteration of neural, endocrine, and immune pathways^46^. We have previously shown that axenic animals lacking microbiota exhibit a consistent increase in dark phase locomotor activity^19^. Using a standard light/dark assay to investigate whether exposure to 0.4 or 1.2 µM E2 caused a colonization status-dependent behavioral response, we showed that only colonized larvae exhibited light phase locomotor hypoactivity. While both colonized and axenic larvae exhibited locomotor hypoactivity in the dark phase, these results are consistent with other studies that demonstrate phase-specific behavioral effects. For example, in other larval zebrafish studies that used similar light/dark assays, developmental exposure to the flame retardants 2,2′,4,4′-tetrabromodiphenyl ether (BDE-47), tricresyl phosphate (TMPP), and phenol, isopropylated, phosphate (3:1) (IPP) caused dark phase-specific hyper- or hypoactivity^47,48^. Developmental 2,2′,4,4′,5-pentabromodiphenyl ether (BDE-99) exposure also resulted in differential phase-specific effects, with hyperactivity observed in the light phase and hypoactivity observed in the dark phase^47^. In addition, Kokel *et al*. showed that embryonic zebrafish exhibit shared and specific behavioral “barcodes” based on their locomotor phenotype in response to a photic stimulus over four different phases following exposure to a large-scale set of drugs^49^. This approach allowed for the classification of putative biological mechanisms of action based on barcode similarity to pharmacological agents with known modes of action. Collectively, these data show that zebrafish light/dark swimming behavior is broadly affected by xenobiotic exposures and that environmental chemicals and drugs produce a diverse set of both phase-shared or phase-specific locomotor effects. This evidence further suggests that changes in light phase-specific locomotor behavior arise from multiple biological pathway-level perturbations. While more work is needed to understand the biology that controls zebrafish swimming behavior in response to light or dark stimuli, the data presented here are in line with previous studies that show differential phase effects of xenobiotic exposures on locomotor activity^47–49^. In addition, our results are the first to show that changes in the stereotypical locomotor response to a light stimulus following exposure to an exogenous environmental chemical are influenced by microbiota.

Microbiota can interact with environmental chemicals via toxicodynamic (alterations in microbial composition or function) or toxicokinetic (microbial xenobiotic biotransformations) mechanisms. In this study, due to logistical challenges associated with our unique concentration-response design, we did not specifically analyze the larval gut microbiome. However, metagenomic analysis revealed that composition of the DMSO vehicle control zebrafish microbiome in this study was similar to that reported from larval gut microbiomes of 10 dpf fish in Stephens *et al*.^37^. Specifically, at the phylum and class levels, microbiota obtained from both whole zebrafish larvae or larval intestinal tract dissections at 10 dpf was dominated by Proteobacteria and Gammaproteobacteria, and contains families that align with core genera identified in adult laboratory and wild zebrafish in Roeselers *et al*. (i.e. Aeromonadaceae, Shewanellaceae, Enterobacteriaceae, and Comamonadaceae)^50^. These similarities are likely because the intestinal tract contains the most bacteria relative to other organs, like in humans^36^. Some differences in composition between microbiota profiles obtained from whole larvae or dissected intestinal tracts were also noted. Namely, the Firmicutes phyla comprised on average 4.2% of all bacterial taxa at 10 dpf in Stephens *et al*. In our study, although we did detect Firmicutes (20/232 OTUs), the relative abundance of this phylum was <1% of all sample totals. At the family level, higher relative abundances of Chromatiaceae were also observed in our study compared with Stephens *et al*. However, higher levels of Chromatiaceae were not observed in all DMSO controls across consecutive experiments done previously in our lab^51^, suggesting that microbiota variability exists within and across aquaculture systems and can be due to a variety of factors including rearing temperature, zebrafish strain, and diet^35,37,38^. Following developmental exposure to five concentrations of E2, global community structure and putative function of zebrafish-associated microbiota were not altered. Microbiota are accustomed to interacting with endogenous E2 and play a significant role in the uptake and metabolism of estrogens^52^. Therefore, it is plausible that exposure to a key endogenous hormone like E2 does not modify the composition of host-associated microbiota in larval zebrafish in the current study. Although one previous study in adult zebrafish using a static-renewal system demonstrated disruption of microbiota following exposure to a nominal concentration of 1 µg/l (3.6 nM) E2^53^, these contrasting results are not surprising given that microbial composition changes with life stage^37^.Taken together, the results presented here suggest that toxicokinetic mechanisms (e.g. microbial-mediated E2 metabolism) may be driving the E2-induced light phase alterations in locomotor activity.

In the current study, measured internal E2 dose varied depending on colonization status. Paradoxically, 10 dpf axenic zebrafish contained significantly more (2.5–3.7 times) parent (unconjugated) E2 compared to conventionally colonized or axenic colonized on day 1 larvae. Non-targeted analysis revealed that peak areas of putative metabolites E2 sulfate and E2 glucuronide were also elevated in axenic larvae, while peak areas of E3 sulfate, E1 sulfate, and E1 glucuronide were similar in colonized and axenic zebrafish. In general, estrogen metabolism occurs primarily in the hepatobiliary system and the intestinal “estrobolome” (the bacterial genes that are capable of metabolizing estrogens)^30^, resulting in potential subsequent detoxification or activation. One explanation for the observed locomotor phenotype following chemical exposure is that E2 is deconjugated and then converted to a more active metabolite, either by microbial enzymes or a more metabolically competent host. Previously published work shows that microbiota bioactivate pharmacological agents like diclofenac, indomethacin, ketoprofen, and sulfasalazine^54,55^. As estrogenic activity is often cell, tissue, model, or endpoint-specific, a more bioactive estrogen profile could be driven by increased deconjugation and bioavailability^56^ or metabolites with enhanced local ER binding or activation^57,58^. For example, sulfonated estrogens, particularly E2 sulfate, can circulate in the blood and reach estrogen target tissues where they may be deconjugated and converted to receptor-active free estrogens^59^. In addition, a study examining the competitive binding activity of dehydroepiandrosterone (DHEA), an abundant steroid hormone that serves as a precursor to estrogen and androgen, and it’s sulfonated metabolite (DHEA-S), showed that DHEA-S, but not DHEA, was competitive with E2 for ERɑ and ERβ binding and stimulated MCF-7 cell proliferation^60^. Parent E2 and E2 glucuronide have also been shown to bind to and activate ERα in the liver^61^. Furthermore, under different physiological conditions, E2 metabolites can differentially activate ERɑ and ERβ signaling pathways, elicit downstream gene activation, and trigger intracellular signaling cascades^62^, which may influence neurodevelopment.

Important limitations of the non-targeted approach used in this study included the inability to detect the parent compound, unknown differences in extraction efficiency, column affinity, breakdown products, ionization efficiency^63^, and a lack of analytical standards for E2 metabolites. While these limitations precluded direct comparisons between levels of E2 metabolites within an exposure group and across colonization statuses, these data support the targeted results and suggest that colonization status influences E2 metabolism. In future follow-up studies, quantification using a targeted approach would allow for more detailed comparisons of E2 metabolite profiles in colonized and axenic zebrafish. It is also possible that E2 metabolites potentially contributing to the observed behavioral effect in colonized larvae in the light phase may not have been identified. For instance, we did not detect hydroxylated E2 metabolites that have been reported in mammals^64^. While more work is needed to enhance detection and determination of E2 metabolites in zebrafish, the application of non-targeted analysis led to the simultaneous identification of five unique sulfonated or glucuronidated E2 metabolites with unknown toxicity profiles that may contribute to colonization-specific alterations in locomotor activity. In addition, chemical toxicokinetics is not simply defined by biotransformation (e.g. bioactivation). As this study did not fully assess absorption, distribution, or excretion of E2 in colonized or axenic zebrafish, other potential mechanisms may also be related to the novel behavioral phenotype observed in the light phase in colonized larvae described here.

Host-associated microbiota are recognized as important modulators of the complex interactions between factors such as diet, sex, race, and life-stage and many human diseases^65^. An increasing body of evidence suggests that microbiota also mediate health effects of xenobiotic exposure including gastrointestinal toxicity and immune responses^66–69^. Our results indicate that microbiota are required for E2-induced light phase locomotor hypoactivity, further supporting the idea that microbiota and chemicals may interact to influence biological processes. This work also raises an interesting point related to hazard identification, suggesting that microbiota may be an important factor for characterizing chemical interactions with a host organism. Continued investigation of how estrogens and other environmental chemicals interact with the microbiota-gut-brain axis may uncover the biological mechanisms by which microbiota communicate with the nervous system and influence brain development and behavior.

## Methods

### Zebrafish husbandry

All experiments involving zebrafish were approved by the Institutional Animal Care and Use Committee at the U.S. EPA National Health and Environmental Effects Research Laboratory and performed in accordance with appropriate guidelines and regulations. A mixed wild type adult zebrafish line (*Danio rerio*) was used for all studies^48^. Adult zebrafish were housed in 6 L tanks (at a density of ~ 8 fish/l) and fed Gemma Micro 300 (Skretting) once daily and shell free E-Z Egg (Brine Shrimp Direct) twice daily during the week. On weekends, zebrafish were fed both food sources once daily. Adults were maintained on a 14 h:10 h light:dark cycle at 28.5 °C. For spawning, 60–100 adults were placed in 10 L angled static breeding tanks overnight. The following morning, adults were transferred to new bottom tanks containing treated reverse osmosis water (60 mg/l sodium bicarbonate and 0.4 g/l Crystal Sea Bioassay Formula Marine Mix) and embryos were collected after 45 min.

### Chemicals

17β-estradiol (E2) was purchased from Sigma Aldrich (#E8875, CAS: 50-28-2; Lot: SLBP6339V). Stock solution aliquots (40 mM) were prepared by dissolving neat chemical into anhydrous dimethyl sulfoxide (DMSO) and stored at −80 °C. For each 10-day experiment, fresh working solutions were prepared by thawing stock solution aliquots and performing serial dilutions in DMSO. Working solutions were stored in 4 ml amber glass vials at room temperature. All exposure groups contained a final concentration of 0.1% DMSO (v/v). Vehicle controls received 0.1% DMSO only. For targeted chemistry, E2-3,4-^13^C_2_ (internal standard, Product#: CLM-803-S; Lot#: SDDI-011) was obtained from Cambridge Isotope Laboratories, Inc. (Tewksbury, MA). Stock solutions were prepared in methanol and acetonitrile and stored at −20 °C. Intermediate standards were prepared fresh daily from stocks. All reagents and solvents were reagent grade or high-performance liquid chromatography (HPLC) grade.

### Exposures

E2 exposures were performed in T25 tissue culture flasks incubated at 26 °C on a 14 h:10 h light:dark cycle. Zebrafish were continuously exposed in a semi-static system from 1 to 10 days post-fertilization (dpf). For all experiments, chemical exposures began on day 1 (Fig. 1). All flasks were housed statically through 6 dpf, followed by daily renewal of chemical exposure solutions in concert with an 80% media change (0.2 µm filter-sterilized 10% Hanks Balanced Salt Solution (FS-10% HBSS) from 6–9 dpf. On days 6–9, each flask also received 75 kGy gamma-irradiated Gemma Micro 75 as a food source at a final concentration of 0.04% (v/v) (Fig. 1). Dead embryos or larvae were removed from each flask during media changes. For the developmental toxicity experiment, conventionally colonized (CC, colonized with aquaculture facility microbiota on day 0) zebrafish were exposed to 0.05–30 µM E2. These concentrations were selected based on previous zebrafish studies and zebrafish assays within the U.S. EPA ToxCast Dashboard (https://actor.epa.gov/dashboard/)^25,70,71^. For 16S rRNA gene sequencing, conventionally colonized zebrafish were exposed to non-teratogenic concentrations ranging from 0.34–3.5 µM E2. For 3-cohort behavior and mass spectrometry, conventionally colonized, axenic colonized on day 1, and axenic zebrafish were exposed to 0.4 or 1.2 µM E2.

### Axenic derivation and conventionalization with fish facility microbiota

Axenic (AX; microbe-free) zebrafish were generated as previously described^19,39,40^ (Fig. 1). Briefly, embryos were resuspended in FS-10% HBSS containing antibiotics (amphotericin B (0.25 μg/ml), kanamycin (5 μg/ml) and ampicillin (100 μg/ml)) for four hours at 26 °C. At 5 hours post fertilization (hpf), embryos were treated in a 15 ml conical tube with 0.5% poly(vinylpyrrolidone)-iodine (PVP-I; CASRN 25655-41-8) for 2 min and 0.05% bleach for 20 min and sorted into sterile T25 tissue culture flasks (15–30 embryos per flask, depending on experiment) in 25 ml of FS-10% HBSS. As a control for the derivation process, a subset of axenic embryos was conventionalized with aquaculture facility microbiota at 1 dpf to generate the axenic colonized on day 1 (AC1) zebrafish cohort. Embryos were conventionalized by 80% media change which included 10 ml fish facility water (FRW, fish room water; 5 µm syringe-filtered) and 10 ml of FS-10% HBSS (Fig. S8). To maintain consistency between cohorts, axenic and conventionally colonized (colonized on day 0, not treated with antibiotics, PVP-I, or bleach) flasks also underwent an 80% media change at 1 dpf. Similar to axenic colonized on day 1 zebrafish, conventionally colonized embryos received 10 ml FRW and 10 ml of FS-10% HBSS. Axenic embryos received 10 ml of 0.2 µm filter-sterilized fish facility water (FS-FRW) and 10 ml of FS-10% HBSS (Fig. S8).

For experiments involving all three zebrafish cohorts, media sterility was tested as previously described^19^. Briefly, at 1 and 10 dpf, two tryptic soy agar (TSA) plates (Sigma, #22091) were inoculated with 10 µl of media from each flask. At 10 dpf, the sterility of media from axenic flasks was further tested by inoculating 100 µL of flask media into tubes of Nutrient Broth (Sigma, #70122), Brain Heart Infusion Broth (Sigma, #53286), or Sabouraud Dextrose Broth (Sigma, #S3306). Plates and tubes were incubated at 26 °C under aerobic and anaerobic conditions for at least seven days. Contaminated flasks were excluded from the study.

### Behavior testing

At 10 dpf, morphologically normal larvae were removed from flasks using a sterile transfer pipet and placed into 48-well plates containing 500 µl of FS-10% HBSS per well. Plates were sealed with Microseal A film (BioRad, #MSA5001), wrapped in Parafilm, and placed in the dark in a temperature controlled behavior testing room at 26 °C, for at least 2 hr prior to testing. For testing, microtiter plates were placed on a Noldus tracking apparatus and locomotor activity was recorded for 40 mins. The light program consisted of a 20-min acclimation period in the dark (0 lux) followed by a 10 min light period (5 lux) and a 10 min dark period (0 lux). All tests were carried out between 11:15 am–4:00 pm. Videos were analyzed using Ethovision software Version 12 (Noldus Information Technology) as described previously^48^.

### DNA sequencing of 16S rRNA gene

Whole-body zebrafish homogenates were used to evaluate changes in microbial community structure and predicted function. DNA extraction and sequencing of the 16S rRNA gene was completed as previously described^19^. Briefly, 10 dpf larvae (n = 4 biological replicates, 10 larvae per replicate) were collected, anesthetized, and homogenized. DNA was isolated from each sample using a ZR-Duet™ DNA/RNA MiniPrep Kit (Zymo Research #D7003). Total DNA yield was quantified and samples were stored at −80 °C. DNA (250 ng) from each sample was added to triplicate PCR reactions along with barcoded primers^72^ specific for the V4 region of the 16S rRNA gene and amplified with the Roche FastStart High Fidelity PCR System (Sigma-Aldrich, #4738292001). PCR reactions were run at 95 °C for 2 min, followed by 25 cycles of 95 °C for 30 sec, 55 °C for 30 sec and 72 °C for 1 min, with a 10 min final extension at 72 °C. Triplicate reactions were pooled and products were purified and normalized with the SequalPrep Normalization Plate Kit (ThermoFisher, #A1051001). Samples were pooled by volume and DNA was sequenced using the Illumina MiSeq Reagent Kit v2 (500 cycles, #MS-102-2003) and Illumina MiSeq instrument. Positive and negative PCR control reactions were run with every 30 samples^19^.

### Analysis of 16S rRNA gene sequences

For microbial community structure and predicted metagenomic function analyses, paired-end sequences were trimmed at a length of 250 base pairs and quality filtered at <0.5% expected error using USEARCH v7^73^. Reads were analyzed using the QIIME 1.9.0 software package^74,75^. The total number of reads for each sample can be found in Table S4. A cutoff of 500 reads per sample was used for downstream analyses. One sample did not meet this criterion (3.5 µM E2_R4). A closed-reference OTU table was generated within QIIME-1.9.0 and used to assess microbial composition and generate PICRUSt (Phylogenetic Investigation of Communities by Reconstruction of Unobserved States) functional predictions^76^ with the Greengenes 13_8 reference database (https://catalog.data.gov). Alpha and beta diversity analyses were performed using PRIMER 7 software (Primer-E v7.0.11) for total number of species, Margalef’s richness index, Pielou’s evenness index, Simpson’s index, Shannon’s diversity index, Bray-Curtis similarities, and non-metric multidimensional scaling (NMDS) ordination plots as previously described^19^. Stacked bar plots were generated using OTUs or predicted functions that contributed at least 1% to any of the sample totals. The KEGG orthology classification scheme was used for functional annotations^77^ and relative abundances of predicted functional pathways were formatted as previously described^78^. Kruskal-Wallis and pairwise Wilcoxon tests were conducted (ɑ = 0.05) using E2 as the main categorical variable. Linear Discriminant Analysis (LDA) scores >2.0 were used to assess functional enrichment. Microbial community structure was also analyzed with mothur-generated OTU tables and the Silva reference database as previously described^19,79^ and provided for future reference as a taxonomic record (Table S5).

### Sample generation and preparation for mass spectrometry

Conventionally colonized, axenic colonized on day 1 and axenic zebrafish larvae were exposed to 0.1% DMSO or 0.4 or 1.2 µM E2 as described above. Blank (fish-free) flasks containing gamma-irradiated Gemma Micro 75 only were included as a control. On day 1, 1 ml of media was collected from all flasks immediately after dosing to measure initial E2 concentrations. At 10 dpf, larvae were transferred from each flask to a vial containing a final volume of 500 µl FS-10% HBSS (n = 2–5 biological replicates, 10 larvae per replicate). Larvae were anesthetized on ice, flash frozen in liquid nitrogen, and stored at −80 °C. Media samples (1 ml) from each flask were also collected at 10 dpf and stored at −80 °C. Larval tissue samples were homogenized in 200 µl of 0.1% formic acid in acetonitrile containing the E2-3,4-^13^C_2_ internal standard using a FastPrep 24 homogenizer (MP Biomedicals, Santa Ana, CA) and ~150 mg, 1.0 mm diameter zirconia/silica beads. The homogenate was centrifuged at 14,000 rpm for 15 minutes at 4 °C. For analysis, 150 µl of the supernatant was diluted in a liquid chromatography (LC) vial with 150 µl of HPLC grade water. Exposure media was prepared for analysis by diluting 200 µl with 50 µl methanol containing the E2-3,4-^13^C_2_ internal standard. Calibration and verification standards were prepared in solvent: 0.1% formic acid in 50% acetonitrile and water for zebrafish tissue samples and 20% methanol in water for exposure media samples. Blanks and quality control samples were prepared by adding appropriate amounts of tissue homogenate or exposure media.

### Targeted mass spectrometry and analysis

Targeted chemistry analysis was conducted by liquid chromatography-tandem mass spectrometry (LC-MS/MS) on an Accela UPLC and TSQ Quantum Ultra triple quadrupole mass spectrometer (Thermo Fisher Scientific, Waltham, MA) equipped with atmospheric pressure photo ionization (APPI) source with krypton lamp operated in positive ionization mode. LC separation was achieved using Kinetex EVO, 50 × 2.1 mm, C18, 2.6 µm, 100 å LC column (Phenomenex, Torrance, CA) with gradient elution at a flow rate of 360 µl/min using A (5% methanol and water) and B (5% water and methanol). Initial LC conditions, 80% A and 20% B, were held for 15 seconds followed by a linear ramp to 100% B at 8 minutes, and held at 100% B for 2 minutes. The column was equilibrated for 2 minutes at initial conditions. Total run time was 12 minutes. Toluene was added to the LC flow post column at 40 µl/min using a PHD Ultra syringe pump (Harvard Apparatus, Holliston, Massachusetts). Source conditions were optimized for the precursor [M-H2O + H]^+^ ions of E2. Collision energy and collision pressure were optimized for product ions of E2 and E2-(3,4-^13^C_2_).

Integration, calibration, and quantitation were performed using Xcalibur 3.0.63 (Thermo Fisher Scientific). The observed measured range in zebrafish tissue was 486 fmole/larva - 15 picomole/larva with a 56 fmole/larva method detection limit (MDL). Recovery of 80% was observed for samples spiked at 1.9 pmol/larva and matrix effects were observed to be less than 10%. The observed range in exposure media was 45.9 nM - 2.31 µM with a 4 nM MDL. 86% recovery was observed for samples spiked at 459 nM and matrix effects were observed to be less than 10%. Batch results were accepted based on the following criteria: 1) The six standard calibration curve had a correlation coefficient >0.99 and accuracy tolerance ≤20%, 2) >75% of all individual quality control standards had sample accuracy tolerance ≤30% and %RSD (precision) ≤20%, and 3) blank response was < MDL.

### Non-targeted mass spectrometry and analysis

To identify endogenous and xenobiotic metabolites associated with E2 exposure and microbial status, non-targeted mass spectrometric analysis was performed within a four-week period on the same samples used for targeted mass spectrometry. Sample extract (10 µl) was injected in triplicate. Analyte separation was accomplished using Waters Acquity UPLC^®^ BEH C_18_ (2.1 × 50 mm, 1.7 µm) connected to an Agilent 1290 Infinity II Liquid Chromatography system (Palo Alto, CA) equipped with a degasser, binary pump, and autosampler. The mobile phase flow rate for gradient elution was set at 200 µl/min using A (aqueous 0.1% formic acid) and B (acetonitrile with 0.1% formic acid). The gradient started with 90% A and 10% B for 2 min, followed by a linear ramp to 100% B after 15 min. This condition was kept for 5 min before returning to starting mobile phase conditions at 21 min. The column was re-equilibrated for the next injection for 9 min. Total run time was 30 min. An Agilent 6530B Accurate-Mass Quadrupole Time-of-Flight Mass Spectrometer (Palo Alto, CA) with a Dual AJS ESI source was operated under positive and negative electrospray ionization in full scan mode (100–1000 m/z). The following spray settings were employed: capillary voltage (±3500 V), nozzle voltage (500 V), fragmentor (135 au), skimmer (65 au), octupole RF peak (750 au), gas temperature (300 °C), gas flow (1 L/min), nebulizer pressure (40 psig), sheath gas temperature (350 °C), and sheath gas flow (11 L/min). Data were acquired under 2 GHz extended dynamic range mode.

Raw data were initially processed using Agilent Profinder vendor software (v.8.00) for molecular feature extraction and integration, with additional filtering using in-house scripts (Supplemental Methods). Chemical features (unique empirical mass-to-charge ratio (m/z) and retention time (min)) were tentatively assigned based on hits against the EPA Chemistry Dashboard (https://comptox.epa.gov/dashboard/) and an Agilent Personal Compound Database and Library (PCDL) of the METLIN database^80^. Monoisotopic ion masses for components of the E2 metabolism pathway (see Fig. 6A) were then separately extracted for each data file using a 10 ppm mass window. The extracted ion chromatograms were smoothed with a 9-point moving average and integrated with Agilent’s Agile 2 algorithm. For samples which lacked an obvious peak, a time window corresponding to the average peak window was manually integrated to capture a noise value. If a sample showed no chromatographic noise at the elution time, the value was recorded as zero.

### Statistical analyses

The AC_50_ value for E2 (i.e. concentration that elicits a 50% inhibitory response) was obtained from the zebrafish developmental toxicity assay using the log(agonist) vs. normalized response–Variable slope equation (GraphPad Prism 7)^71^. A one-way analysis of variance (ANOVA) with Tukey pairwise comparisons was used for analysis of the zebrafish developmental toxicity assay. An analysis of similarities (ANOSIM) test (concentration = ordered factor) was used to assess changes in microbial composition. For comparisons of Bray-Curtis similarity scores, a permutational one-way analysis of variance PERMANOVA with pairwise Monte Carlo comparisons was used (p < 0.05). Alpha diversity metrics were analyzed using a Kruskal-Wallis and Dunn’s pairwise multiple comparisons test (p < 0.05). All behavior data were analyzed using SAS v9.4 software using a mixed effects repeated measures model. Each individual fish was used as a subject for the repeated measures, with the movement measures across phase (10 min light or 10 min dark period) and time (five time points within each phase, reflecting each two min period). Plate, flask and experiment effects were tested as random factors, found to be not significant (ɑ = 0.05), and subsequently removed from the model. Main effects of each fixed factor (i.e. concentration, phase, or time), and any interaction between or among the factors, were tested within each status. Backwards stepwise elimination was used to identify the most parsimonious model. If a significant 3-way interaction (concentration*phase*time, p < 0.05) or 2-way interaction (concentration*phase, p < 0.05) was observed, differences between concentration groups were tested in each phase using t-tests with a Tukey-Kramer adjustment for multiple comparisons.

For targeted chemistry data analysis, multiple linear regression models were used to identify significant predictors of E2 tissue concentration (pmole/larva) or E2 exposure media concentration (µM). Backwards stepwise elimination was used to identify the most parsimonious model using square root-adjusted values to satisfy modeling assumptions related to normality and homoscedasticity. The effects of concentration, status, or interaction on zebrafish tissue or exposure media concentrations or abundance were assessed (p < 0.05). For zebrafish tissue, any negative values (five non-detects, only found in DMSO controls) were assigned the lowest non-negative value (0.0003 pmol/larva) in the dataset. If a significant interaction between dose and status was observed, pairwise comparisons across groups were evaluated using differences of least squares means and Bonferroni-adjusted p-values (p < 0.05). For non-targeted chemistry analysis, Spearman rank correlation coefficients were calculated to assess the relationships between integrated peak areas for each detected E2 metabolite and measured E2 tissue concentrations from targeted analysis.

## Supplementary information

Dataset S1

Dataset S2

## Data Availability

The datasets generated during the current study are available by searching for the manuscript title at https://catalog.data.gov.

## References

[1] Thursby E, Juge N (2017). Introduction to the human gut microbiota. Biochem J.

[2] Ursell LK (2014). The intestinal metabolome: an intersection between microbiota and host. Gastroenterology.

[3] Kline KA, Falker S, Dahlberg S, Normark S, Henriques-Normark B (2009). Bacterial adhesins in host-microbe interactions. Cell Host Microbe.

[4] Gunther C, Josenhans C, Wehkamp J (2016). Crosstalk between microbiota, pathogens and the innate immune responses. Int J Med Microbiol.

[5] Sharon G, Sampson TR, Geschwind DH, Mazmanian SK (2016). The Central Nervous System and the Gut Microbiome. Cell.

[6] Wheeler R, Chevalier G, Eberl G, Gomperts Boneca I (2014). The biology of bacterial peptidoglycans and their impact on host immunity and physiology. Cell Microbiol.

[7] Choi JJ (2013). Exercise attenuates PCB-induced changes in the mouse gut microbiome. Environ Health Perspect.

[8] Van de Wiele T (2010). Arsenic Metabolism by Human Gut Microbiota upon *in Vitro* Digestion of Contaminated Soils. Environ Health Persp.

[9] Van de Wiele T (2005). Human colon microbiota transform polycyclic aromatic hydrocarbons to estrogenic metabolites. Environ Health Persp.

[10] U.S. EPA, America’s Children and the Environment: Neurodevelopmental Disorders (2015).

[11] Kelly, J. R., Minuto, C., Cryan, J. F., Clarke, G. & Dinan, T. G. Cross Talk: The Microbiota and Neurodevelopmental Disorders. *Front Neurosci-Switz* **11**, 10.3389/fnins.2017.00490 (2017). 10.3389/fnins.2017.00490 PMC5605633 28966571

[12] Carding S, Verbeke K, Vipond DT, Corfe BM, Owen LJ (2015). Dysbiosis of the gut microbiota in disease. Microb Ecol Health Dis.

[13] Braniste V (2014). The gut microbiota influences blood-brain barrier permeability in mice. Sci Transl Med.

[14] Hoban AE (2016). Regulation of prefrontal cortex myelination by the microbiota. Transl Psychiatry.

[15] Ogbonnaya ES (2015). Adult Hippocampal Neurogenesis Is Regulated by the Microbiome. Biol Psychiatry.

[16] Bercik Premysl, Denou Emmanuel, Collins Josh, Jackson Wendy, Lu Jun, Jury Jennifer, Deng Yikang, Blennerhassett Patricia, Macri Joseph, McCoy Kathy D., Verdu Elena F., Collins Stephen M. (2011). The Intestinal Microbiota Affect Central Levels of Brain-Derived Neurotropic Factor and Behavior in Mice. Gastroenterology.

[17] Davis DJ, Bryda EC, Gillespie CH, Ericsson AC (2016). Microbial modulation of behavior and stress responses in zebrafish larvae. Behav Brain Res.

[18] Davis DJ (2016). Lactobacillus plantarum attenuates anxiety-related behavior and protects against stress-induced dysbiosis in adult zebrafish. Sci Rep.

[19] Phelps, D. *et al*. Microbial colonization is required for normal neurobehavioral development in zebrafish. *Sci Rep-Uk* **7**, 10.1038/s41598-017-10517-5 (2017). 10.1038/s41598-017-10517-5 PMC5593827 28894128

[20] Bergman A (2013). The Impact of Endocrine Disruption: A Consensus Statement on the State of the Science. Environ Health Persp.

[21] Mccarthy MM (2008). Estradiol and the developing brain. Physiol Rev.

[22] Bondesson M, Hao R, Lin CY, Williams C, Gustafsson JA (2015). Estrogen receptor signaling during vertebrate development. Bba-Gene Regul Mech.

[23] Arambula SE, Jima D, Patisaul HB (2018). Prenatal bisphenol A (BPA) exposure alters the transcriptome of the neonate rat amygdala in a sex-specific manner: a CLARITY-BPA consortium study. Neurotoxicology.

[24] Brion François, Le Page Yann, Piccini Benjamin, Cardoso Olivier, Tong Sok-Keng, Chung Bon-chu, Kah Olivier (2012). Screening Estrogenic Activities of Chemicals or Mixtures In Vivo Using Transgenic (cyp19a1b-GFP) Zebrafish Embryos. PLoS ONE.

[25] Saili KS (2012). Neurodevelopmental low-dose bisphenol A exposure leads to early life-stage hyperactivity and learning deficits in adult zebrafish. Toxicology.

[26] Adlercreutz H, Pulkkinen MO, Hamalainen EK, Korpela JT (1984). Studies on the role of intestinal bacteria in metabolism of synthetic and natural steroid hormones. J Steroid Biochem.

[27] Kornman KS, Loesche WJ (1982). Effects of estradiol and progesterone on Bacteroides melaninogenicus and Bacteroides gingivalis. Infect Immun.

[28] Baker JM, Al-Nakkash L, Herbst-Kralovetz MM (2017). Estrogen-gut microbiome axis: Physiological and clinical implications. Maturitas.

[29] Fuhrman BJ (2014). Associations of the Fecal Microbiome With Urinary Estrogens and Estrogen Metabolites in Postmenopausal Women. J Clin Endocr Metab.

[30] Plottel CS, Blaser MJ (2011). Microbiome and Malignancy. Cell Host Microbe.

[31] Lescak, E. A. & Milligan-Myhre, K. C. Teleosts as Model Organisms To Understand Host-Microbe Interactions. *J Bacteriol* **199**, 10.1128/JB.00868-16 (2017). 10.1128/JB.00868-16 PMC5512220 28439034

[32] Rawls JF, Samuel BS, Gordon JI (2004). Gnotobiotic zebrafish reveal evolutionarily conserved responses to the gut microbiota. Proc Natl Acad Sci USA.

[33] Howe K (2013). The zebrafish reference genome sequence and its relationship to the human genome. Nature.

[34] Sumbre G, de Polavieja GG (2014). The world according to zebrafish: how neural circuits generate behavior. Front Neural Circuits.

[35] Brugman S (2016). The zebrafish as a model to study intestinal inflammation. Dev Comp Immunol.

[36] Sender R, Fuchs S, Milo R (2016). Revised Estimates for the Number of Human and Bacteria Cells in the Body. PLoS Biol.

[37] Stephens WZ (2016). The composition of the zebrafish intestinal microbial community varies across development. Isme J.

[38] Rawls JF, Mahowald MA, Ley RE, Gordon JI (2006). Reciprocal gut microbiota transplants from zebrafish and mice to germ-free recipients reveal host habitat selection. Cell.

[39] Milligan-Myhre K (2011). Study of Host-Microbe Interactions in Zebrafish. Zebrafish: Disease Models and Chemical Screens, 3rd Edition.

[40] Pham LN, Kanther M, Semova I, Rawls JF (2008). Methods for generating and colonizing gnotobiotic zebrafish. Nat Protoc.

[41] Jones HE, Conrad HS (1933). The growth and decline of intelligence: a study of a homogeneous group between the ages of ten and sixty. Genet. Psychol. Monogr..

[42] Wallace M, Luine V, Arellanos A, Frankfurt M (2006). Ovariectomized rats show decreased recognition memory and spine density in the hippocampus and prefrontal cortex. Brain Research.

[43] Morgan MA, Pfaff DW (2001). Effects of estrogen on activity and fear-related behaviors in mice. Horm Behav.

[44] Steimer T (2002). The biology of fear- and anxiety-related behaviors. Dialogues Clin Neurosci.

[45] Locci A (2017). Neonatal estradiol exposure to female rats changes GABAA receptor expression and function, and spatial learning during adulthood. Horm Behav.

[46] Collins SM, Surette M, Bercik P (2012). The interplay between the intestinal microbiota and the brain. Nat Rev Microbiol.

[47] Glazer L (2018). Developmental exposure to low concentrations of two brominated flame retardants, BDE-47 and BDE-99, causes life-long behavioral alterations in zebrafish. Neurotoxicology.

[48] Jarema KA, Hunter DL, Shaffer RM, Behl M, Padilla S (2015). Acute and developmental behavioral effects of flame retardants and related chemicals in zebrafish. Neurotoxicol Teratol.

[49] Kokel D (2010). Rapid behavior-based identification of neuroactive small molecules in the zebrafish. Nat Chem Biol.

[50] Roeselers G (2011). Evidence for a core gut microbiota in the zebrafish. ISME J.

[51] Catron TR (2019). Host Developmental Toxicity of BPA and BPA Alternatives Is Inversely Related to Microbiota Disruption in Zebrafish. Toxicol Sci.

[52] Adlercreutz H (1976). Intestinal metabolism of estrogens. J Clin Endocrinol Metab.

[53] Chen L (2018). Dysregulation of Intestinal Health by Environmental Pollutants: Involvement of the Estrogen Receptor and Aryl Hydrocarbon Receptor. Environ Sci Technol.

[54] Peppercorn MA, Goldman P (1972). The role of intestinal bacteria in the metabolism of salicylazosulfapyridine. J Pharmacol Exp Ther.

[55] Saitta KS (2014). Bacterial beta-glucuronidase inhibition protects mice against enteropathy induced by indomethacin, ketoprofen or diclofenac: mode of action and pharmacokinetics. Xenobiotica.

[56] Raftogianis, R., Creveling, C., Weinshilboum, R. & Weisz, J. Estrogen metabolism by conjugation. *J Natl Cancer Inst Monogr*, 113–124 (2000). 10.1093/oxfordjournals.jncimonographs.a024234 10963623

[57] Barnea ER, MacLusky NJ, Naftolin F (1983). Kinetics of catechol estrogen-estrogen receptor dissociation: a possible factor underlying differences in catechol estrogen biological activity. Steroids.

[58] Lustig RH, Mobbs CV, Pfaff DW, Fishman J (1989). Temporal actions of 16 alpha-hydroxyestrone in the rat: comparisons of lordosis dynamics with other estrogen metabolites and between sexes. J Steroid Biochem.

[59] Song WC (2001). Biochemistry and reproductive endocrinology of estrogen sulfotransferase. Ann N Y Acad Sci.

[60] Miller KK (2013). DHEA metabolites activate estrogen receptors alpha and beta. Steroids.

[61] Barosso IR (2012). Sequential activation of classic PKC and estrogen receptor alpha is involved in estradiol 17ss-D-glucuronide-induced cholestasis. Plos One.

[62] Toran-Allerand CD (1992). Estrogen receptors colocalize with low-affinity nerve growth factor receptors in cholinergic neurons of the basal forebrain. Proc Natl Acad Sci USA.

[63] Khedr A, Alandal AM (2016). Liquid chromatography-tandem mass spectrometric analysis of ten estrogen metabolites at sub-picogram levels in breast cancer women. J Chromatogr B.

[64] Lee AJ, Kosh JW, Conney AH, Zhu BT (2001). Characterization of the NADPH-dependent metabolism of 17beta-estradiol to multiple metabolites by human liver microsomes and selectively expressed human cytochrome P450 3A4 and 3A5. J Pharmacol Exp Ther.

[65] Hollister EB, Gao CX, Versalovic J (2014). Compositional and Functional Features of the Gastrointestinal Microbiome and Their Effects on Human Health. Gastroenterology.

[66] Cho Youngji, Abu-Ali Galeb, Tashiro Hiroki, Kasahara David I., Brown Traci A., Brand Jeffrey D., Mathews Joel A., Huttenhower Curtis, Shore Stephanie A. (2018). The Microbiome Regulates Pulmonary Responses to Ozone in Mice. American Journal of Respiratory Cell and Molecular Biology.

[67] Hubbard TD (2017). Dietary Broccoli Impacts Microbial Community Structure and Attenuates Chemically Induced Colitis in Mice in an Ah receptor dependent manner. J Funct Foods.

[68] Wallace BD (2015). Structure and Inhibition of Microbiome beta-Glucuronidases Essential to the Alleviation of Cancer Drug Toxicity. Chem Biol.

[69] Wallace BD (2010). Alleviating cancer drug toxicity by inhibiting a bacterial enzyme. Science.

[70] Baker Nancy, Knudsen Thomas, Williams Antony (2017). Abstract Sifter: a comprehensive front-end system to PubMed. F1000Research.

[71] Padilla S (2012). Zebrafish developmental screening of the ToxCast (TM) Phase I chemical library. Reprod Toxicol.

[72] Kozich JJ, Westcott SL, Baxter NT, Highlander SK, Schloss PD (2013). Development of a Dual-Index Sequencing Strategy and Curation Pipeline for Analyzing Amplicon Sequence Data on the MiSeq Illumina Sequencing Platform. Appl Environ Microb.

[73] Edgar RC (2013). UPARSE: highly accurate OTU sequences from microbial amplicon reads. Nat Methods.

[74] Caporaso JG (2010). QIIME allows analysis of high-throughput community sequencing data. Nat Methods.

[75] Caporaso JG (2011). Global patterns of 16S rRNA diversity at a depth of millions of sequences per sample. P Natl Acad Sci USA.

[76] Langille MGI (2013). Predictive functional profiling of microbial communities using 16S rRNA marker gene sequences. Nat Biotechnol.

[77] Kanehisa M, Goto S (2000). KEGG: Kyoto Encyclopedia of Genes and Genomes. Nucleic Acids Res.

[78] Segata Nicola, Izard Jacques, Waldron Levi, Gevers Dirk, Miropolsky Larisa, Garrett Wendy S, Huttenhower Curtis (2011). Metagenomic biomarker discovery and explanation. Genome Biology.

[79] Schloss PD (2009). Introducing mothur: Open-Source, Platform-Independent, Community-Supported Software for Describing and Comparing Microbial Communities. Appl Environ Microb.

[80] Smith CA (2005). METLIN - A metabolite mass spectral database. Ther Drug Monit.

