# Supplementary material for: Microbiota alter metabolism and mediate neurodevelopmental toxicity of 17β-estradiol
Source: Sci Rep. 2019 May 8;9:7064. doi: 10.1038/s41598-019-43346-9 (PMC6506524; doi:10.1038/s41598-019-43346-9)
Supplement: Supplementary file 1 — Dataset S1 [file 41598_2019_43346_MOESM1_ESM.docx]

**Microbiota alter metabolism and mediate neurodevelopmental toxicity of 17β-estradiol**

Tara R. Catron^1^, Adam Swank^2^, Leah Wehmas^3^, Drake Phelps^1^, Scott P. Keely^4^, Nichole E. Brinkman^4^, James McCord^1^, Randolph Singh^1^, Jon Sobus^5^, Charles E. Wood^6^, Mark Strynar^5^, Emily Wheaton^4^ and Tamara Tal^3^

^1^Oak Ridge Institute for Science and Education, Oak Ridge, TN; ^2^U.S. EPA/ORD/NHEERL/RCU, RTP, NC; ^3^U.S. EPA/ORD/NHEERL/ISTD, RTP, NC; ^4^U.S. EPA/ORD/NERL/SED, Cincinnati, OH; ^5^U.S. EPA/ORD/NERL/EMMD, RTP, NC; ^6^Boehringer Ingelheim, Ridgefield, CT.

***Address Correspondence:** Tamara Tal, Integrated Systems Toxicology Division, National Health and Environmental Effects Research Laboratory, U.S. EPA, 109 T.W. Alexander Drive, B105-03, Research Triangle Park, North Carolina 27711 (Email: [tal.tamara@epa.gov](mailto:tal.tamara@epa.gov); Tel: 919-541-0506).

**Keywords:** microbiome, 17β-estradiol, zebrafish, developmental toxicity

**Supplemental Methods**

**SAS code for behavior data: comparison of DMSO controls**

*****************************************************************************

***Study: MB93_113

***Program: MB93_113dmso.sas

***check that CC, AC1 and AX controls act similarly in the light, and that AX is different

***(more movement) from CC and AC1 in the dark.

******************************************************************************

options nocenter pageno=1 ls=140 ps=45;

ods noresults;

run;

libname in 'hard drive location here’

%let expt=MB93_113;

%let cmpd=E2;

title1 "Experiment: &expt Compound: &cmpd";

title2 'QA2: check activity by status for dmso controls';

data a;

set in.mb93_113rptd;

if compound='DMSO';

run;

proc mixed data=a ratio covtest;

class fishid status phase time;

model movemnt = status phase phase*time status*phase status*phase*time /ddfm=kr ;

repeated phase time*phase/subject=fishid(status) type=un@ar(1) rcorr;

title3 'Full Model';

run;

***if status*phase*time pvalue<0.05 then run by phase;

proc sort data=a;

by phase;

proc mixed data=a ratio covtest;

by phase;

class fishid status time plate flask;

model movemnt = status time status*time /ddfm=kr ;

repeated time/subject=fishid(status) type=ar(1) rcorr;

lsmeans status/pdiff adjust=tukey;

title3 'status*phase*time interaction, run separately by phase';

run;

***if status*phase*time pvalue>=0.05 then remove from model;

proc mixed data=a ratio covtest;

class fishid status phase time;

model movemnt = status phase phase*time status*phase /ddfm=kr ;

repeated phase time*phase/subject=fishid(status) type=un@ar(1) rcorr;

slice status*phase/sliceby=phase pdiff adjdfe=row adjust=tukey;

title3 'No status*phase*time interaction, remove from model';

run;

**SAS code for behavior data: analysis of CC, AC1, and AX larval behavior**

****************************************************************************

***Study: MB93_113

***Program: MB93_113analysis.sas

***Separately for CC, AC1 and AX data, test for any effect of dose wrt light and dark

*****************************************************************************

options nocenter pageno=1 ls=140 ps=45;

ods noresults;

run;

libname in 'L:\Lab\NHEERL_Tal_Lab\Statistics\MB93_113_E2 behavior\sasdata';

%let expt=MB93 or MB113;

%let cmpd=E2;

title1 "Experiment: &expt Compound: &cmpd";

title2 'Analysis of dose effect over time by status';

data a;

set in.mb93_113rptd;

proc sort data=a; by status;

proc mixed data=a ratio covtest;

by status;

class fishid dose phase time;

model movemnt = dose phase phase*time dose*phase dose*phase*time/ddfm=kr;

repeated phase time*phase/subject=fishid(dose) type=un@ar(**1**) rcorr;

run;

***if pvalue for dose*phase*time>=0.05 then remove from model;

proc mixed data=a ratio covtest;

by status;

*where status='1CC' '2AC1' '3AX'; ***choose correct status;

where status in ('2AC1'); ***choose correct status;

class fishid dose phase time plate flask;

model movemnt = dose phase phase*time dose*phase/ddfm=kr;

repeated phase time*phase/subject=fishid(dose) type=un@ar(1) rcorr;

slice dose*phase/sliceby=phase pdiff adjdfe=row adjust=tukey;

run;

***if pvalue for dose*phase*time < 0.05 run separately by phase;

proc sort data=a;

by status phase;

proc mixed data=a ratio covtest;

by status phase;

*where status='1CC' '2AC1' '3AX'; ***choose correct status;

where status='2AC1'; ***choose correct status;

class fishid dose time;

model movemnt = dose time dose*time/ddfm=kr;

repeated time/subject=fishid(dose) type=ar(1) rcorr;

lsmeans dose/pdiff adjdfe=row adjust=tukey;

run;

**Non-targeted chemistry and analysis**

Raw data was processed using Agilent Profinder vendor software (v.8.00) for molecular feature

extraction and integration using default settings with a few modifications. The initial data processing codes can be found at <https://github.com/jpmccord/MPP-Filtering-R-Workflow-for-NTA>. Chemical features were tentatively assigned based on formula hits against the EPA Chemistry Dashboard(https://comptox.epa.gov/dashboard/) and an Agilent Personal Compound Database and Library (PCDL) of the METLIN database1. Chemical features and assignments were filtered using an inhouse data processing script in R using analytical criteria as follows: A feature was removed entirely if: (1) no treatment group exhibited that feature in at least 80% of its sample replicates with a CV <30%; and (2) the maximum abundance for that feature (across all treatment groups) was below 100,000 counts. A chemical feature was removed from an individual treatment group if the feature was not detected in >80% of sample replicates with a CV <50%, and a maximum abundance of >10,000 counts. A subset of the filtered features was selected for binary comparison across dose and colonization status.

Between-group comparison of 1.2 μM E2-exposed vs. DMSO controls and 1.2 μM E2-exposed

CC or AC1 cohorts vs. AX cohort were made and a shortlist of features exhibiting > 2-fold abundance difference within any comparison were selected (94 features). Dose and status comparisons were tested using a Mann-Whitney U test with multiple hypothesis correction using

a Benjamini-Hochberg FDR2 of 5%. Features of interest were selected using threshold cutoff of 2- fold change and adjusted p-value < 0.05.

**References**

1. Smith CA, O'Maille G, Want EJ, Qin C, Trauger SA, Brandon TR, et al. 2005. Metlin - a

metabolite mass spectral database. Ther Drug Monit 27:747-751.

2. Benjamini Y and Hochberg Y. 1995. Controlling the False Discovery Rate: A Practical and

Powerful Approach to Multiple Testing. J R Stat Soc Series B Stat Methodol.57: 289-300.

**Non-targeted chemistry analysis in-house data processing script for feature filtering and preparation of Figures S5 and S6**

check.packages <- function(package){

new.package <- package[!(package %in% installed.packages()[, "Package"])]

if (length(new.package))

install.packages(new.package, dependencies = TRUE)

sapply(package, require, character.only = TRUE)

}

packages<-c("tidyverse", "stringr", "sqldf", "readxl", "dtplyr")

check.packages(packages)

mapping <- read_excel("Sample Mapping.xlsx", sheet = 2) %>%

unique()

rep_counts <- mapping %>%

group_by(Prep) %>%

summarize(count = sum(!is.na(Reps)))

mymetdata <- read_csv("Merged_Estradio_Mpp.csv") %>%

mutate(X4B_subMedAbun = ifelse(is.na(X4B_subMedAbun), X4b_subMedAbun, X4B_subMedAbun)) %>%

select(-X4b_subMedAbun) %>%

mutate(Compound = paste0(Mass,"@",RetentionTime)) %>%

gather(contains("subMed"), key = zcolname, value = abun) %>%

left_join(mapping) %>%

unique()%>%

filter(!is.na(Dose))

filtered <- mymetdata %>%

select(Compound, zcolname, abun, Prep, Reps, Mass, RetentionTime) %>%

group_by(Compound,Prep) %>%

summarize(mean = mean(abun, na.rm = TRUE),

sd = sd(abun, na.rm = TRUE),

n = sum(as.numeric(!is.na(abun))),

CV = sd/mean) %>%

left_join(mymetdata) %>%

left_join(rep_counts) %>%

select(Compound,mean,sd,n,CV,count,Mass,RetentionTime,Prep,Colonization,Dummy_Col,Dose) %>%

unique()

low_freq_feature<- filtered %>%

mutate(freq = n/count) %>%

select(Compound, freq) %>%

group_by(Compound) %>%

summarize(max_freq = max(freq)) %>%

filter(max_freq >= 0.8)

high_CV_feature <- filtered %>%

select(Compound, CV) %>%

group_by(Compound) %>%

summarize(minCV = min(CV, na.rm = TRUE),

maxCV = max(CV, na.rm = TRUE),

meanCV = mean(CV, na.rm = TRUE)) %>%

filter(minCV <= 0.3)

low_abun_feature <- filtered %>%

select(Compound,Prep,mean) %>%

mutate(abun_flag = mean < 100000) %>%

mutate(mean = ifelse(abun_flag == FALSE, mean, NA)) %>%

group_by(Compound) %>%

summarize(sum.mean = mean(mean, na.rm = TRUE)) %>%

filter(sum.mean > 1000)

low_freq_prep<- filtered %>%

mutate(freq = n/count) %>%

select(Compound, Prep, freq) %>%

group_by(Compound,Prep) %>%

summarize(max_freq = max(freq)) %>%

mutate(freq_flag = max_freq > 0.8)

low_abun_prep <- filtered %>%

select(Compound,Prep,mean) %>%

mutate(abun_flag = mean > 10000)

high_CV_prep <- filtered %>%

select(Compound, Prep, CV) %>%

group_by(Compound, Prep) %>%

summarize(minCV = min(CV, na.rm = TRUE),

maxCV = max(CV, na.rm = TRUE),

meanCV = mean(CV, na.rm = TRUE)) %>%

mutate(CV_flag = meanCV < 0.5)

CV_filtered <- mymetdata %>%

filter(Compound %in% low_freq_feature$Compound,

Compound %in% high_CV_feature$Compound,

Compound %in% low_abun_feature$Compound) %>%

left_join(low_freq_prep) %>%

left_join(low_abun_prep) %>%

left_join(high_CV_prep) %>%

mutate(abun = ifelse(freq_flag == FALSE, NA, abun)) %>%

mutate(abun = ifelse(CV_flag == FALSE, NA, abun))

effects_filter <- CV_filtered %>%

group_by(Compound,Prep) %>%

summarize(mean = mean(abun, na.rm =TRUE)) %>%

mutate(mean = ifelse(is.na(mean), 1, mean)) %>%

spread(key = Prep, value = mean) %>%

mutate(a = `4`/`6`,

b = `7`/`9`,

c = `10`/`12`,

d = `6`/`9`,

e = `9`/`12`,

f = `6`/`12`)

dose.ratio <- select(effects_filter, a, b, c, d, e, f) %>%

gather(-Compound, key = comparison, value = ratio) %>%

summarize(max = abs(max(log10(ratio))),

min = abs(min(log10(ratio))),

scale = ifelse( max > min, max, min)) %>%

filter(scale > .3)

effects_filter <- effects_filter %>%

filter(Compound %in% dose.ratio$Compound)

wide_filtered <- CV_filtered %>%

filter(Compound %in% dose.ratio$Compound) %>%

select(Compound, IonMode, Mass, RetentionTime, zcolname, abun) %>%

spread(zcolname, value= abun) %>%

left_join(select(mymetdata,Compound,contains("Annotations")))%>%

rename( RT = RetentionTime,

`Compound Name` = Compound)%>%

mutate(`CAS ID` = c(""),

Formula = ifelse(grepl("$[A-Z]",`Compound Name`), `Compound Name`, NA)) %>%

unique()

master_annotations <- wide_filtered %>% select(`Compound Name`, contains("Annotations"))

write_excel_csv(wide_filtered, "/EE2Shortlist.csv", na = "")

write_excel_csv(mapping, "samplemappings.csv")

filelist <- c(

"AC1 High v AC1 DMSO.xlsx",

"AX High v AX DMSO.xlsx",

"CC High v CC DMSO.xlsx",

"AC1 High v AX High.xlsx",

"CC High v AX High.xlsx",

"CC High v AC1 High.xlsx"

)

getMPPdata <- function(filepath) {

file <- read_excel(filepath)

file[10] <- (file[3] < 0.05 & file[4] > 1.5)

file["color"] <- file[5]

file <- file %>%

mutate(color = ifelse(V10 == TRUE, color, "grey")) %>%

filter(!is.na(color))

return(file)

}

figuresources <- lapply(filelist, getMPPdata)

highlight.features <- c()

plotme <- function(source,xrow, yrow, mycolor) {

p <- ggplot() +

theme_bw()+

geom_hline( yintercept = -log10(.05), linetype = 3) +

geom_vline( xintercept = -log(1.5,2), linetype = 2) +

geom_vline( xintercept = log(1.5,2), linetype = 2) +

theme(panel.grid.major = element_blank(),

panel.grid.minor = element_blank(),

axis.line = element_line(colour = "black"),

plot.title = element_text(hjust = 0.5),

#legend.title = element_blank(),

legend.position = "bottom",

panel.background = element_blank())+

geom_point(data = source, aes( y= -log10(source[[yrow]]), x = source[[xrow]], color = source[[mycolor]]), size = 3)+

scale_color_manual(name = "",

values=c("blue","grey","red"),

labels = c("Down","NA","Up"))

return(p)

}

graphs <- list()

for (i in 1:6) {

graphs[[i]] <- plotme(figuresources[[i]], 9, 3, "color")+

labs( title = gsub("High","1.2 uM E2",str_extract(filelist[i],".*(?=\\.xlsx)")),

x = "log2(Fold Change)",

y = "-log10(p-value)")

}

for (i in 1:length(graphs)) {

tiff(file = paste0(gsub("\\|","-",graphs[[i]]$labels$title),ifelse(grepl("Q",graphs[[i]]$labels$y)," Q"," p"),".tiff"),

res = 300,

width = 5,

height = 5,

units = "in",

compression = "lzw")

print(graphs[[i]])

dev.off()

}

for (i in 1:length(graphs)) {

holdingcell <- figuresources[[i]]

output <- tibble(c(1:nrow(holdingcell)))

output["Compound Name"] <- holdingcell[1]

output["Mann-Whitney p-value"] <- holdingcell[2]

output["BH Correct p-value"] <- holdingcell[3]

output["log2(Fold-Change)"]<-holdingcell[6]

output["color"]<-holdingcell["color"]

output <- output %>%

mutate(color = ifelse(color %in% highlight.features,NA,color)) %>%

select(-color)

output[output == "grey"] <- NA

output <- left_join(output, master_annotations) %>%

filter(`BH Correct p-value` < 0.05) %>%

select(-`c(1:nrow(holdingcell))`)

write_excel_csv(output, na = "", path=paste0(gsub("\\|","-",graphs[[i]]$labels$title),".csv"))

}

**List of Supplemental Figures**

**Figure S1.** Bray-Curtis similarities following E2 exposure.

**Figure S2.** Alpha diversity metrics following E2 exposure.

**Figure S3.** Level 3 predicted KEGG functions for differentially abundant predictions.

**Figure S4.** Axenic zebrafish exhibit dark phase hyperactivity relative to colonized control zebrafish.

**Figure S5.** Initial non-targeted analysis workflow.

**Figure S6.** Within status results for non-targeted analysis.

**Figure S7.** Between status results for non-targeted analysis.

**Figure S8.** Experimental design workflow depicting new conventionalization technique.


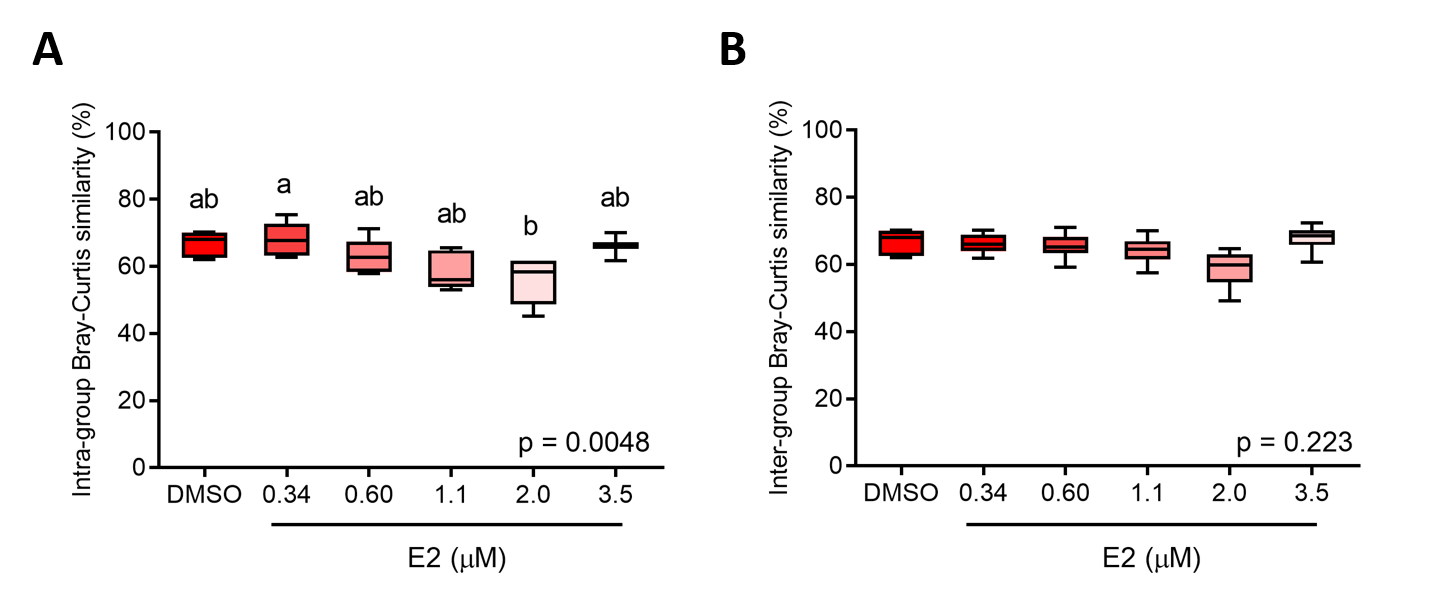


**Figure S1. Bray-Curtis similarity scores (%) between treatment groups.** Bray-Curtis similarities for each chemical concentration were compared to DMSO vehicle controls (i.e. inter-group Bray-Curtis similarity). DMSO vehicle control similarity scores are also shown. PERMANOVA p-value is reported on each graph. A PERMANOVA with Monte Carlo pairwise comparisons statistics were used to determine significance (p < 0.05). n = 4 biological replicates with 10 larvae per replicate.


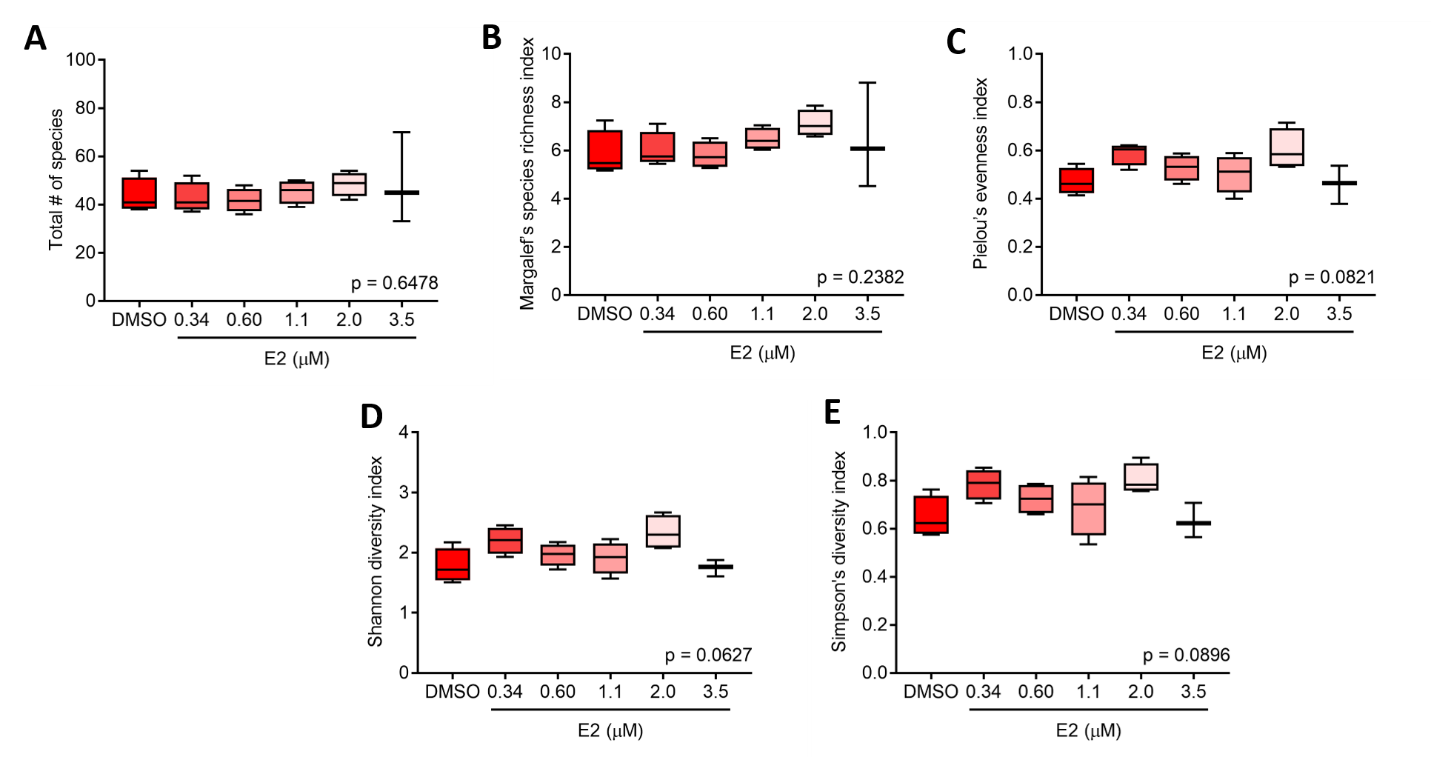
**Figure S2. Alpha diversity metrics for E2 exposure.** 16S rRNA gene sequencing was performed on 10 dpf zebrafish exposed to E2. **(A)** Total number of species, **(B)** Margalef’s species richness index, **(C)** Species evenness, **(D)** Shannon diversity index, and **(E)** Simpson’s diversity are shown. n=4 biological replicates with 10 larvae per replicate. Kruskal-Wallis non-parametric test (p<0.05) followed by Dunn’s pairwise comparisons (p<0.05) was used to determine significance.


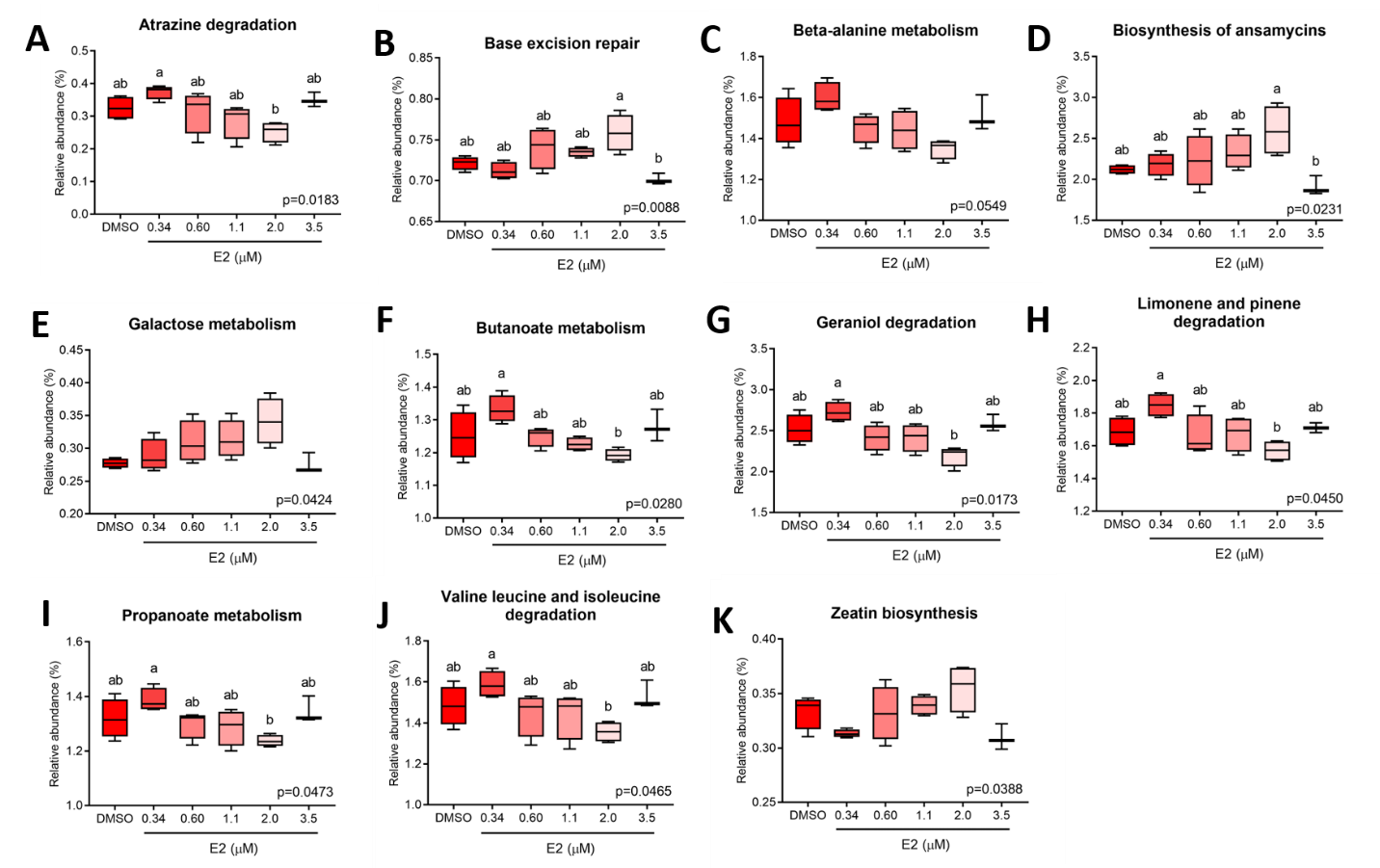


**Figure S3. E2 exposure does not impact predicted Level 3 KEGG functions. (A-K)** Boxplots for all significantly enriched (LEfSe analysis) Level 3 KEGG predictions are shown. Kruskal-Wallis non-parametric test (p < 0.05) followed by Dunn’s pairwise comparisons test (p<0.05) was used to determine significance.

**
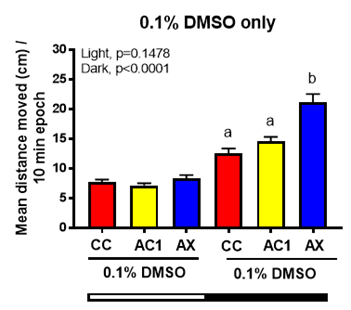
**

**Figure S4. Axenic zebrafish exhibit dark phase hyperactivity relative to colonized zebrafish**. To directly compare baseline activity levels in DMSO-exposed axenic, conventionally colonized, and axenic colonized on day 1 zebrafish, control data from Figure 4A-I was analyzed to show the mean distance moved during each 10 min light or dark period. If a significant 3- or 2-way interaction was observed using a linear mixed effect repeated measures model, subsequent Tukey pairwise comparisons were made. Different letters indicate significant differences in the dark phase (p < 0.05). (n=37-56).

**
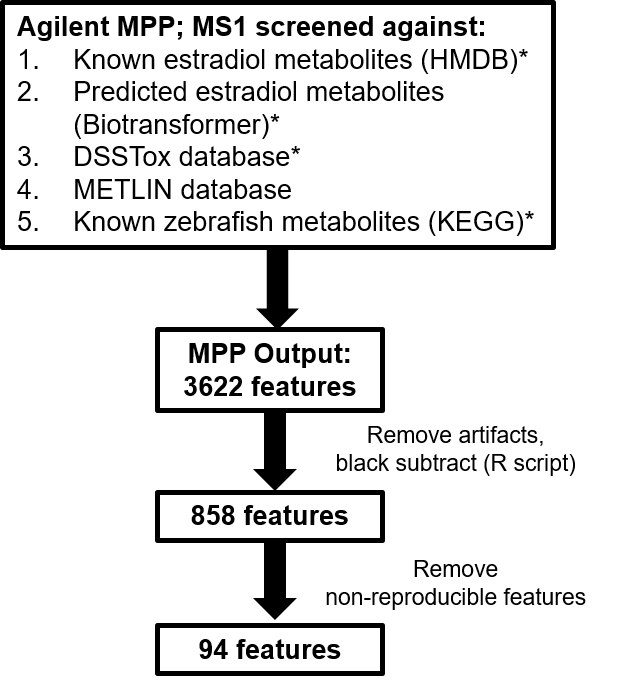
**

**Figure S5.** **Initial workflow describing molecular feature extraction criteria.** A non-targeted screening approach was initially used to analyze zebrafish cellular lysate to try to identify E2 metabolites associated with microbial colonization status. *Databases were searched, but did not identify any additional molecular features that were not found in the METLIN database.

**
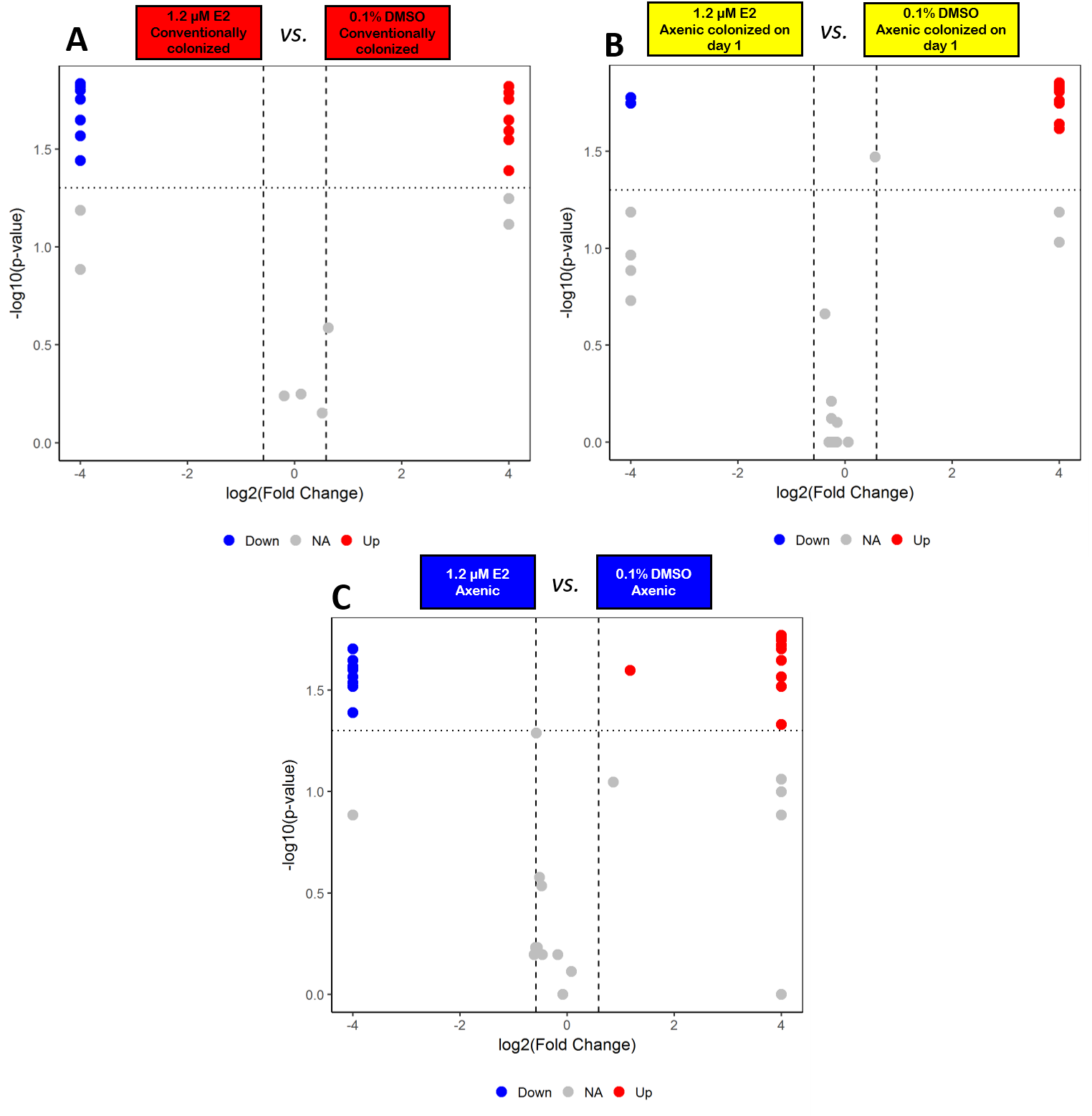
**

**Figure S6**. **Molecular features that were differentially expressed across concentration for a given colonization status.** Zebrafish cellular lysate used for targeted E2 analysis was analyzed by a non-targeted screening approach to identify metabolites associated with E2 exposure and microbial colonization status. Within-group comparisons were performed on 10 dpf larvae exposed to 1.2 µM E2 or 0.1% DMSO. Volcano plots depicting (A) 1.2 µM E2 conventionally colonized *vs*. 0.1% DMSO conventionally colonized, (B) 1.2 µM E2 axenic colonized on day 1 *vs*. 0.1% DMSO axenic colonized on day 1 and (C) 1.2 µM E2 axenic *vs.* 0.1% DMSO axenic comparisons are shown. Blue dots = down regulated features. Red dots = upregulated features. n=4-5 (10 larvae per biological replicate).

**
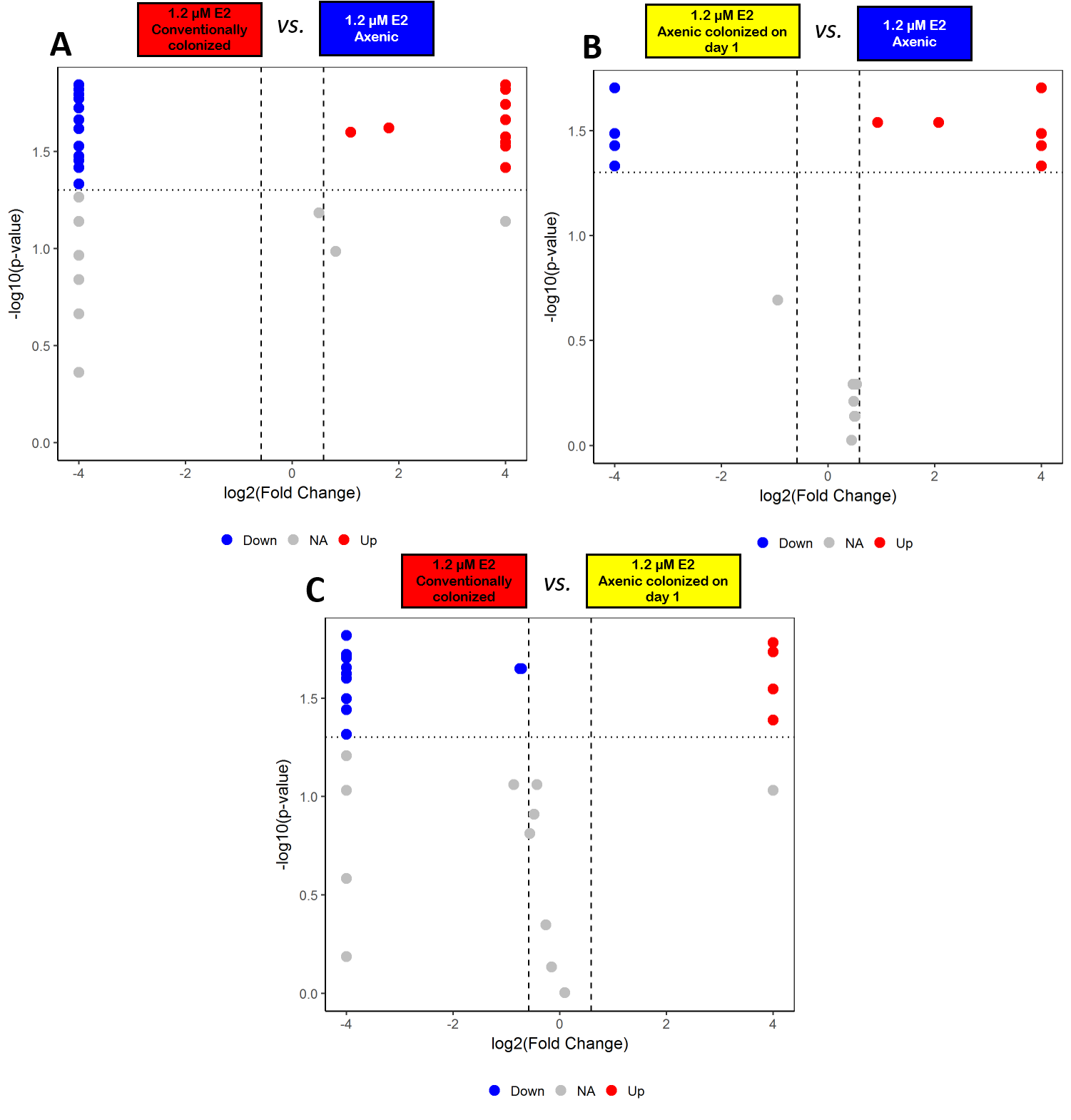
**

**Figure S7. Features that were differentially expressed across colonization status when exposed to 1.2 µM E2.** Zebrafish cellular lysate used for targeted E2 analysis was analyzed by a non-targeted screening approach to identify metabolites associated with E2 exposure and microbial colonization status. Between-group comparisons were performed on 10 dpf larvae exposed to 1.2 µM E2. Volcano plots depicting (A) conventionally colonized *vs*. axenic, (B) axenic colonized on day 1 *vs*. axenic, or (C) conventionally colonized *vs.* axenic colonized on day 1 comparisons are shown. Blue dots = down regulated features. Red dots = upregulated features. n=2-5 (10 larvae per biological replicate).

**
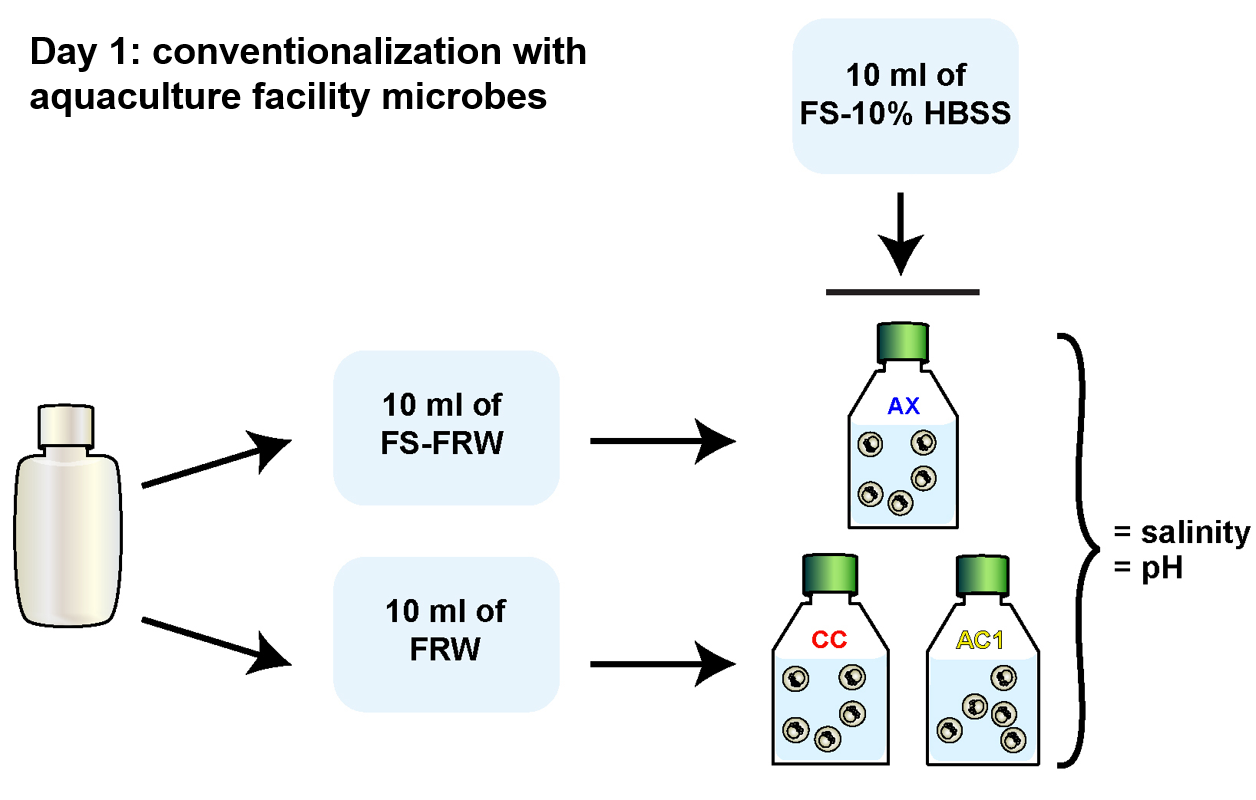
Figure S8. Conventionalization design.** New conventionalization technique for adding microbes to all three zebrafish cohorts on day 1 is shown. FS = filter-sterilized. FRW = fish room water. HBSS = 10% Hanks Balanced Salt Solution. Zebrafish cohorts: CC = conventionally colonized, AC1 = axenic colonized on day 1, AX = axenic.
